# CEBPB-Regulated Gastric Cell Plasticity Promotes Liver Metastasis of Gastric Cancer

**DOI:** 10.34133/cancomm.0016

**Published:** 2026-03-12

**Authors:** Zhixiang Zuo, Jiaqi Liang, Li-Na He, Kai Xu, Muren Hu, Kunming Zhang, Wei Gao, Junyi Yin, Lanlin Zhang, Boning Ma, Zhiqian Hu, Pengfei Zhang, Hong Jiang

**Affiliations:** ^1^ State Key Laboratory of Oncology in South China, Cancer Center, Collaborative Innovation Center for Cancer Medicine, School of Life Sciences, Sun Yat-sen University, Guangzhou 510060, Guangdong, P. R. China.; ^2^ Department of Pathology, Sun Yat-sen Memorial Hospital, Sun Yat-sen University, Guangzhou 510300, Guangdong, P. R. China.; ^3^ Department of General Surgery, Tongji Hospital, School of Medicine, Tongji University, Shanghai 200092, P. R. China.; ^4^ Department of Gastrointestinal Surgery, the First Affiliated Hospital, Zhejiang University School of Medicine, Hangzhou 310003, Zhejiang, P. R. China.; ^5^ Department of Oncology, The First Affiliated Hospital, Jinan University, Guangzhou 510630, Guangdong, P. R. China.; ^6^ Department of General Surgery, Shanghai General Hospital, Shanghai Jiao Tong University School of Medicine, Shanghai 200080, P. R. China.; ^7^ Department of Cancer Center, Tongji Hospital, Tongji University School of Medicine, Shanghai 200065, P. R. China.; ^8^ Department of Medical Oncology, Zhongshan Hospital, Fudan University, Shanghai 200032, P. R. China.; ^9^ Zhongshan School of Medicine, Sun Yat-sen University, Guangzhou 510080, Guangdong, P. R. China.; ^10^ Department of Medical Oncology, Shanghai Geriatric Medical Center, Shanghai 201104, P. R. China.

## Abstract

**Background:** Liver metastasis represents the most common distant dissemination in gastric cancer (GC) but persists as a challenging condition to manage, and its driving molecular mechanisms remain poorly understood. This study aimed to uncover the key regulatory drivers of GC liver metastasis and explore their potential as therapeutic targets. **Methods:** Herein, we employed a multifaceted approach combining single-cell RNA sequencing, bulk transcriptomics, epigenomics analyses of GC primary tumors and normal adjacent tissues, paired liver metastasis, and circulating tumor cells, alongside in vitro and in vivo experimental validation, to investigate how metastatic GC cells spread to and adapt within the liver microenvironment. **Results:** We discovered that GC cells undergoing liver metastasis transcriptionally reprogrammed into a high plasticity state. This plasticity was mediated by the transcription factor CCAAT enhancer-binding protein beta (*CEBPB*), which activated liver metastasis-associated genes through enhancer reprogramming. Notably, *CEBPB*-driven reprogramming enhanced the metastatic potential of GC cells and enabled them to evade immune surveillance via interactions between cluster of differentiation 155 (CD155) and T cell immunoreceptor with Ig and ITIM domains (TIGIT). Blocking the CD155–TIGIT interplay inhibited liver metastasis and restored T cell cytotoxicity. **Conclusions:** Our study identifies CEBPB-mediated transcriptional and epigenetic reprogramming as a fundamental driver of GC liver metastasis. Our findings underscore the *CEBPB*/*CD155*/*TIGIT* axis as a promising therapeutic target for liver-metastatic GC.

## Background

Gastric cancer (GC) is one of the most prevalent malignancies of the digestive system and ranks as the fifth leading cause of cancer-related mortality worldwide, with approximately 970,000 new cases and 660,000 deaths reported in 2022 [1]. The management of advanced-stage GC, characterized by high invasiveness and poor prognosis, remains a major clinical challenge [2,3]. The liver is the most common target organ for hematogenous metastasis in GC [4]. While systemic chemotherapy remains the primary treatment option for gastric cancer liver metastasis (GCLM), emerging modalities such as trans-arterial therapy, radio-frequency ablation, targeted therapies, and immunotherapies have provided alternative options [5–7]. Nevertheless, the prognosis of GCLM remains challenging. Therefore, it is crucial to further investigate the mechanisms underlying GCLM and develop improved interventions.

GCLM is a multistep process and involves complicated biological events, including the aberrant expression of biomolecules in GC cells, abnormal transduction of relevant signaling pathways, and interactions between cancer cells and various resident cells in the liver tumor microenvironment (TME) [4,5]. Although some clinical studies have delved into GCLM [8], its underlying regulatory mechanisms remain largely unexplored, and no specific targets for disease progression are currently available in the clinic. Recent efforts have underlined the crucial roles of cancer cell plasticity and disease heterogeneity in promoting liver metastasis [9–11]. Furthermore, single-cell profiling tools, such as single-cell RNA sequencing (scRNA-seq), have evolved to revolutionize the appreciation of cellular plasticity, tumor heterogeneity, and diverse functional states that shape the intricate tumor ecosystem [12,13]. Previous single-cell studies designed for GC mainly focused on premalignant and primary tumor samples, with few or no metastatic lesions being investigated [14–19]. In addition, the dynamic interplay between cancer cells and other TME cells is underappreciated due to the lack of holistic profiling of TME components [17,18]. A comprehensive decoding of the primary tumors and paired liver metastases is warranted to uncover the drivers of liver metastasis and to track how these metastatic “seeds” adapt to and colonize in the new liver microenvironment.

In this study, we performed scRNA-seq analysis on GC primary tumors and their normal adjacent tissues (NATs), paired liver metastases, and circulating tumor cells (CTCs), and integrated other publicly available GC scRNA-seq datasets to portray the comprehensive transcriptional dynamics of GCLM. We identified metastasis-related malignant cells showing high plasticity, which is regulated by CCAAT enhancer-binding protein beta (*CEBPB*)-mediated enhancer reprogramming. These GC malignant cells with high plasticity evaded the surveillance of the immune system through interactions with CD8^+^ T cells via the immune checkpoint cluster of differentiation 155 (CD155)–T cell immunoreceptor with Ig and ITIM domains (TIGIT). Interrupting the interplay restrained GC development and metastasis via restoring CD8^+^ T cell cytotoxicity. Our findings offer novel insights into the cellular and molecular mechanisms underlying GCLM and suggest potential strategies for combating this challenging aspect of GC.

## Materials and Methods

### Human samples

This study was approved by the Ethics Committee of Zhongshan Hospital of Fudan University (Shanghai, China; Approval No. B2023-289R), and we complied with all relevant ethical regulations. The patients described in this study for scRNA-seq (in-house cohort #1, *n* = 11) and for further immunohistochemistry (IHC) staining validation (in-house cohort #2, *n* = 20) were provided written informed consent (Table S1).

### Mouse model

BALB/c nude mice and 615-strain mice were purchased from Beijing SiPeiFu Biotechnology Co., Ltd. (Beijing, China) and SiLaiKe Laboratory Animal Co., Ltd. (Shanghai, China), respectively. The animals were housed in facilities under specific pathogen-free (SPF) conditions, and food and water were provided ad libitum. All animal experiments in this study were performed in accordance with protocols approved by the Institutional Animal Care and Use Committee (IACUC) of Medical Discovery Leader Co., Ltd. (Hebei, China; Approval No. MDL2023-08-28-01). All animal experiments were conducted in accordance with the *Guide for the Care and Use of Laboratory Animals* [20]. Before the experiments, all purchased mice were allowed to acclimate to housing conditions for 1 week.

### Cell lines and culture conditions

The human embryonic kidney cell line HEK-293 and human gastric adenocarcinoma cell lines NCI-N87 and SNU-1 were purchased from the Shanghai Cell Bank of the Chinese Academy of Sciences (Shanghai, China). Human gastric adenocarcinoma cell lines MKN-74 and SNU-719, as well as the mouse forestomach carcinoma cell line Mouse Forestomach Carcinoma (MFC), were purchased from SUNNCELL Co., Ltd. (Wuhan, China). All cells were cultured aseptically in Dulbecco,s Modified Eagle Medium (DMEM; Cat. #C11995500BT, Gibco) with 10% fetal bovine serum (FBS) at 37 °C in a humidified incubator with 5% CO_2_.

### Lentivirus production and cell transfection

The short hairpin RNAs (shRNAs) targeting *CEBPB* and *CD155* were synthesized by Shanghai GenePharma Co., Ltd. (Shanghai, China) and subcloned into the pLentiLox 3.7 (pLL 3.7) vector for lentivirus production. Two shRNAs targeted against *CEBPB* (sh*CEBPB* #1, 5′-GCCGCCGCCTGCCTTTAAATC-3′; sh*CEBPB* #2, 5′-GCCCTGAGTAATCGCTTAAAG-3′) and 2 shRNAs targeted against *CD155* (sh*CD155* #1, 5′-GCATGTCTCCTATTCAGCTTT-3′; sh*CD155* #2, 5′-AGTCAGCTGTGACACATGCTT-3′) were used. CEBPB and CD155 overexpression (OE) plasmids were both purchased from Shanghai GenePharma Co., Ltd. Target cDNA was subcloned into the pLenti-CMV-RFP-Puro vector. Recombinant lentiviral vector particles were produced by tri-transfection of the third-generation lentiviral packaging construct into HEK-293 cells according to the manufacturer’s protocol. Viral supernatants were harvested 48 and 72 h posttransfection and used for cancer cell infection. Stable cell populations were selected using 2 μg/ml puromycin (Cat. #P9620, Sigma-Aldrich). Knockdown and overexpression efficiencies were validated by real-time quantitative polymerase chain reaction (qPCR) and Western blotting.

### Clone formation experiments

Cells from each group were digested using trypsin and resuspended at a concentration of 1 × 10^3^ cells/ml. The cell suspension was then seeded into 6-well plates for the colony formation assay. The culture medium was replaced every 3 days, and the cell clones were observed after 14 days of cultivation. Each condition was performed in 3 replicates.

### Transwell assay

Matrigel (Cat. #082724, Abwbio) was diluted in serum-free DMEM at a ratio of 1:8. A total of 100 μl of the diluted Matrigel was added vertically to the upper chamber of the Transwell, evenly spread on the bottom, and then placed in an incubator for 3 h to allow Matrigel to polymerize into the gel matrix. Cells were cultured in DMEM supplemented with 0.1% FBS, and 2 × 10^5^ cells were seeded in the upper chamber of the Transwell chamber with (invasion assay) or without (migration assay) Matrigel. Meanwhile, 500 μl of DMEM containing 10% FBS was added to the lower chamber of the Transwell. To minimize the confounding effect of cell proliferation mediated by CEBPB [21,22] on invasion and migration assays, after incubation for 24 h, the invaded or migrated cells were fixed with paraformaldehyde for 20 min, stained with 0.5% crystal violet staining solution for 10 min, and counted under a microscope.

### Western blotting analysis

Cells were lysed on ice for 30 min. Following lysis, the tube containing cell lysate was centrifuged at 16,000 ×*g* for 10 min to obtain total cellular protein. Total protein concentration was determined using the bicinchoninic acid assay. The protein samples were prepared for sodium dodecyl sulfate–polyacrylamide gel electrophoresis (SDS-PAGE) by denaturing the proteins and mixing them with loading buffer. The prepared samples were loaded into the polyacrylamide gel, and the protein components were separated using SDS-PAGE. Subsequently, the proteins were transferred to polyvinylidene difluoride (PVDF) membrane, which was blocked with 5% skimmed milk for 1 h. The membrane was washed once with Tris-buffered saline with Tween 20 Detergent (TBST) buffer and incubated with primary antibodies against CEBPB or CD155 for 24 h at 4 °C. Following primary antibody incubation, the membrane was washed 3 times with TBST buffer and incubated with the horseradish peroxidase (HRP)-conjugated anti-rabbit IgG secondary antibody for 2 h at room temperature (Table S2). After incubation, the secondary antibody was removed, and the membrane was washed 3 times with TBST buffer. Enhanced chemiluminescence solution was applied to the PVDF membrane, and the protein bands were visualized using the iBright gel imaging system (Thermo Fisher Scientific). The membrane was then subjected to stripping and reprobing for glyceraldehyde-3-phosphate dehydrogenase (GAPDH; Cat. #ab8245, Abcam). Briefly, the membrane was washed 3 times with TBST buffer then placed in the stripping solution (Cat. #21059, Thermo Fisher Scientific) and incubated with agitation for 15 min at 37 °C, and then washed 3 times with TBST buffer. Subsequently, the membrane was blocked with 5% skimmed milk for 1 h, incubated with primary antibodies against GAPDH and the HRP-conjugated anti-mouse IgG secondary antibody for 2 h at room temperature, and then imaged as described above.

### Coculture of CD8^+^ T cells with tumor cells

CD8^+^ T cells sorted from peripheral blood mononuclear cells (PBMCs) were stimulated with cluster of differentiation 3 (CD3)/cluster of differentiation 28 (CD28) activator beads at a ratio of 1:1 and expanded in DMEM complete medium containing 10% FBS, 1% penicillin dual antibiotic, and 100 U/ml interleukin-2 (IL-2). After 24 h of culture, NCI-N87 and MKN-74 cells were transfected with sh*CD155* or sh*CEBPB* lentivirus, while SNU-1 and SNU-719 cells were transfected with CD155-OE or CEBPB-OE lentivirus. Disturbed cells were treated with or without the addition of 500 ng/ml anti-green fluorescent protein (GFP) monoclonal antibodies (mAbs), 50 ng/ml anti-CD155 mAbs (NTX-1088), or 500 ng/ml anti-TIGIT mAbs (Tiragolumab; Table S2). These tumor cells were labeled with Vybrant DiO (Cat. #C1038, Beyotime Biotechnology) for 8 min and resuspended in cell culture medium at a concentration of 2 × 10^5^ cells/ml. The labeled tumor cells were seeded into 96-well plates at 50 μl/well and incubated overnight. Meanwhile, CD8^+^ T cells were collected and resuspended in cell culture medium at a concentration of 1 × 10^6^ cells/ml. For all coculture experiments, CD8^+^ T cells were added to 96-well plates containing tumor cells at varying effector-to-target (E:T) ratios of 1:1, 2:1, 5:1, and 10:1, respectively. After 4 h of coculture, supernatants were removed, and Accutase (Cat. #A1110501, Thermo Fisher Scientific) was added to the wells. The cells were incubated at 37 °C for 10 min to assess the viability of cancer cells. After 8 days of coculture, CD8^+^ T cells were stimulated using CD3 activator, and the levels of interferon γ (IFN-γ) and perforin in the culture supernatant were measured using enzyme-linked immunosorbent assay (ELISA). The assays were performed using the IFN-γ assay kit (Cat. #E-EL-H0108, Elabscience) and the perforin assay kit (Cat. #JL23588, JiangLai Biological), following the manufacturer’s instructions.

### Enhancer luciferase reporter assays

The candidate enhancer sequence of *CEBPB* was amplified by PCR from genomic DNA of the GC cell line NCI-N87. Nhe I and Hind III restriction enzyme sites were incorporated into the 5′ and 3′ ends of the primers, respectively (forward strand: 5′-AGCTAGCGATTGCCTGGCTCTACTCCG-3′; reverse strand: 5′-AAAGCTTGTTTATTGCAGAACTTGTAG-3′). The PCR products and the pGL3-enhancer plasmid were digested with Nhe I (Cat. #HY-KE7027, MedChemExpress) and Hind III (Cat. #HY-KE7018, MedChemExpress) restriction enzymes, respectively. After gel extraction, the candidate *CEBPB* enhancer sequence was ligated into the pGL3-enhancer plasmid and verified by Sanger sequencing.

### IHC assays

The formalin-fixed paraffin-embedded tissue blocks were sectioned into 4-μm-thick slides and baked in an oven at 65 °C for 1 h. Xylene, graded concentrations of ethanol, phosphate-buffered saline (PBS), and 1 mmol/l ethylenediaminetetraacetic acid (EDTA) were used sequentially for deparaffinization, rehydration, and antigen retrieval of the sections. The slides were then incubated with the primary antibody against human CEBPB or mouse Cebpb at 37 °C for 45 min (Table S2). To enhance the signal, the sections were treated with HRP (Cat. #KIT-9921, MXB Biotechnologies) for 15 min at room temperature. Afterward, the slides were washed 3 times with PBS and incubated with an HRP-conjugated anti-rabbit IgG secondary antibody at 37 °C for 15 min. 3,3′-diaminobenzidine (Cat. #ab103723, Abcam) was used as the chromogen and incubated for 5 min at room temperature. Finally, the slides were counterstained with hematoxylin, mounted with neutral balsam, and prepared for observation. The IHC staining was evaluated qualitatively by 3 independent pathologists blinded to the clinical data. The overall staining intensity was categorized into 4 grades: negative (0), weak (1), moderate (2), and strong (3). In cases of scoring discrepancies among the pathologists, a consensus was reached through joint review using a multihead microscope.

### Multiplex immunofluorescence assays

The paraffin sections were dewaxed in xylene, followed by rehydration in a series of ethanol dilutions. Sections were then placed in a box filled with EDTA antigen retrieval buffer (pH 8.0) in a microwave oven for 15 min for antigen repair. Endogenous peroxidase activity was blocked using the peroxidase-blocking solution (Cat. #TT-0851, MXB Technologies) for 10 min. After blocking with 5% bovine serum albumin at room temperature for 30 min, the first primary antibody was added, and the slides were incubated overnight at 4 °C. The HRP-conjugated anti-rabbit IgG secondary antibody was added and incubated at room temperature for 30 min. Fluorophore-conjugated tyramide signal amplification (TSA) was added (Cat. #A40002450, Thermo Fisher Scientific) and incubated at room temperature for 10 min. The slides were then placed in a box filled with EDTA buffer (pH 8.0) in a microwave oven for 16 min (high power for 1 min and low power for 15 min) for antibody stripping. Slides were rinsed with PBS washing buffer (pH 7.4) after each step using a decolorization shaker. Steps from primary antibody incubation to antibody stripping were repeated until all markers were labeled. To counterstain the nuclei, 4′,6-diamidino-2-phenylindole (DAPI) staining solution was applied and incubated in the dark at room temperature for 10 min. Finally, the slides were sealed with an anti-fluorescence quench sealer and imaged under a fluorescence microscope. The primary antibodies and corresponding fluorophore-conjugated TSAs employed were listed as follows and detailed in Table S2: for human liver-metastasis samples, anti-pan-cytokeratin (anti-pan-CK; Aluora 555), anti-CD8 (Aluora 488), anti-TIGIT (Aluora 594), and anti-CD155 (Aluora 647); for mouse liver-metastasis samples, anti-CD8 (Aluora 488) and anti-IFN-γ (Aluora 594).

### Animal experiments and bioluminescence imaging

We constructed orthotopic xenograft nude mouse models to investigate the role of *CEBPB* in liver metastasis of GC. The BALB/c nude mice (*n* = 20) were randomly divided into 4 groups: the NCI-N87 shCtrl group, the NCI-N87 sh*CEBPB* group, the SNU-1 negative control group, and the SNU-1 CEBPB-OE group. Six- to eight-week-old BALB/c nude mice were subjected to intragastric injection with either 1 × 10^7^ cells/ml NCI-N87 shCtrl or NCI-N87 sh*CEBPB* cells or 5 × 10^6^ cells/ml SNU-1 or SNU-1 CEBPB-OE cells. The intragastric injection procedure was performed in accordance with the methodology described in a previous study [23]. Briefly, cancer cells for transplantation were suspended in sterile DMEM supplemented with 10% FBS. Mice were anesthetized using a small animal anesthesia system supplied with oxygen (2 L/min) and isoflurane. A 5-mm incision was then made in the skin overlying the mid-abdomen, and the stomach was exposed. Then, the cancer cells were injected into the serous side of the stomach. The skin incision was closed with an absorbable suture. Mice were monitored for tumor growth on days 3, 14, 21, 28, 35, 42, and 50 by an in vivo bioluminescence imaging system (VISQUE, Invivo Smart-LF, Korea) with substrate excitation (50 mg/ml D-Luciferin potassium salt; Cat. #50227, Sigma-Aldrich). The tumor volumes were calculated using the formula: volume = length × width^2^ × 0.52. To capture CTCs, 0.1 ml of venous blood was collected from the tail veins of mice on days 3, 14, 28, and 50, further processed as detailed in the “CTC isolation” section. On day 50, all mice were sacrificed, and organs were photographed against a white background to observe tumor distribution macroscopically.

For the orthotopic homograft model of GC, 615-strain mice were subjected to intragastric injection with 1 × 10^7^ cells/ml MFC or MFC CEBPB-OE cells. The mice (*n* = 20) were randomly divided into 4 groups: the healthy control group (without cancer cell transplantation), the negative control group (with MFC transplantation), the CEBPB-OE group, and the CEBPB-OE plus anti-TIGIT mAb treatment group. For the CEBPB-OE plus anti-TIGIT mAb treatment group, 100 μg/mouse anti-TIGIT mAb (clone 1G9; Cat. #BE0274, BioXCell) was intraperitoneally administered twice a week from week 3 to week 6 posttransplantation. The tumors were imaged using a bioluminescence imaging system as described above. On day 50, 0.2 ml of venous blood was collected from the tail veins of mice. CD45^+^ cells, CD8^+^ T cells, and NK cells were isolated using the CD45^+^ cell (Cat. #480027, BioLegend), CD8^+^ T cell (Cat. #MIM003N, Elabscience), and NK cell (Cat. #480049, BioLegend) isolation kit according to the manufacturer’s protocol from the mouse plasma, and the proportions of CD8^+^ T cells and NK cells were then determined by flow cytometry. Levels of IFN-γ and tumor necrosis factor-α (TNF-α) in mouse serum were determined by the IFN-γ and TNF-α detection kit (Cat. #JL10967 and #JL10484, JiangLai Biological), as per the manufacturer’s instructions. All mice were sacrificed, and organs were photographed against a white background to observe tumor distribution macroscopically. Visceral specimens of the mice were fixed with paraformaldehyde, embedded in paraffin, and processed for hematoxylin and eosin (H&E) staining and IHC staining.

### CTC isolation

The preparation of immunoliposomes (IMLs) was performed using the reverse-phase evaporation method, as described in a previously published procedure [24]. Briefly, 5 mg of dioleoylphosphocholine (Avanti) and 5 mg of cholesterol were added to a 50-ml 3-neck flask. After ethanol was removed, 1.0 mg of Fe_3_O_4_-hydrophobic magnetic nanoparticles dissolved in 3.0 ml of CH_2_Cl_2_ was transferred into the flask. The mixture in the flask was sonicated on ice. Simultaneously, 2 mg of anti-Vimentin antibody (Cat. #ab20346, Abcam)-modified glycidyl hexadecyl dimethylammonium chloride (Vi-GHDC) was dissolved in 6 ml of ddH_2_O and gradually added to this flask. The preparation of Vi-GHDC was performed according to our previously described method [25]. After emulsification, rotary evaporation was used to remove the residual CH_2_Cl_2_ from the emulsion. The solution was magnetically separated and washed 3 times, and anti-Vimentin-modified IMLs (Vi-IMLs) were obtained. Similarly, human anti-epithelial cell adhesion molecule (EpCAM) antibody (Cat. #ab223582, Abcam) was used for the construction of modified IMLs (Ep-IMLs) for CTC isolation from human samples, and mouse anti-EpCAM antibody (Cat. #ab71916, Abcam) was used for CTC isolation from mouse samples. The detailed preparation process and reagent consumption are described in a previous study [26].

For isolation and identification of CTCs from clinical blood samples, 7.5 ml of venous blood was collected from patients (Table S1) at the specified time point and placed into an EDTA anticoagulant tube. After gentle mixing, the samples were stored in a refrigerator at 4 °C and transported to the laboratory of the CTC Test Center [Jukang (Shanghai) Biotechnology Co., Ltd., Shanghai, China] within 24 h. The CTCs in the peripheral blood were then enriched and screened by the self-made immunomagnetic spheres. First, 10 μl of Ep-IMLs was added to the sample and incubated at room temperature for 15 min, with mixing performed once every 5 min. After the incubation, the centrifuge tube was inserted into the magnetic separator for 10 min to allow adsorption, after which the supernatant was discarded. Next, 10 μl of Vi-IMLs was added to the remaining sample and incubated under the same conditions (15 min at room temperature, mixed every 5 min). After incubation, the tube was again placed in a magnetic separator for 10 min, and the supernatant was discarded. To wash the captured CTCs, 1 ml of PBS was added, and magnetic separation was performed twice. Subsequently, the enriched CTC samples were stained by adding 20 μl of fluorescein isothiocyanate (FITC)-labeled anti-pan-cytokeratin antibody (pan-CK-FITC; Cat. #LY0010, Lieyuan BioPharm), 20 μl of DAPI staining solution, and 20 μl of PE-labeled anti-CD45 antibody (CD45-PE; Cat. #DH0024, AmyJet Scientific). The mixture was incubated for 15 min in the dark. After staining, 1 ml of ddH_2_O was added for 2 rounds of washing, and the sample was resuspended in 20 μl of ddH_2_O. For visual examination, the prepared mixture was evenly smeared onto a poly-lysine-coated anti-slip slide and allowed to air-dry naturally. The droplets were then observed under a fluorescence microscope (OLYMPUS BX61; Olympus Corp) for CTC identification and counting. The criteria for valid CTCs were cells that were pan-CK^+^, DAPI^+^, and CD45^−^. After that, CTCs were counted by a polychromatic fluorescent Cell Counter (BD FACS caliber; Becton Dickinson).

Blood samples were collected from the tail veins of mice to isolate and identify CTCs. CTCs were then isolated using IML incubation followed by magnetic separation. Following a similar protocol to the above CTC recovery method, the supernatant was carefully transferred into a centrifuge tube, and an equal volume of PBS was added and mixed. Self-prepared Vi-IMLs and Ep-IMLs were then used to enrich and capture CTCs in the blood samples. The captured CTCs were subsequently examined under a fluorescence microscope and quantified using a polychromatic fluorescent cell counter.

### Full-length scRNA-seq for CTCs

CTCs were flow-sorted into 96-well plates preloaded with cell lysis buffer. The cell lysis buffer was prepared with 1 μl of 10 mmol/l dNTP mixture (Cat. #R0192, Thermo Fisher Scientific), 1 μl of 10 μmol/l dT oligonucleotide primer (Integrated DNA Technologies), 1.9 μl of 1% Triton X-100 (Cat. #T8787-50ML, Sigma-Aldrich), and 0.1 μl of 40 U/μl RNA polymerase inhibitor (Cat. #2313B, Takara). The buffer was stored at −80 °C until use. To lyse the cells, the lysis buffer with single cells was removed from −80 °C, heated at 72 °C for 3 min, and then kept at 4 °C before adding the reverse transcription master mix. The SMART-seq2 protocol [27] was used to generate single-cell cDNA and libraries. The reverse transcription step followed the SMART-seq2 protocol exactly, using a 5′-biotinylated template switching oligo (5′-AAGCAGTGGTATCAACGCAGAGTACATrGrG+G-3′, Qiagen). The first round of 24-cycle PCR was performed with 10 μl of reverse transcription product using a KAPA HiFi PCR Kit (Cat. #KK2101, Roche Diagnostics) according to the manufacturer’s protocol. PCR products were cleaned up with a 0.8:1 ratio of Ampure XP beads (Cat. #A63881, Beckman Coulter). The quality of single-cell cDNA was assessed on the TapeStation system (Agilent) with the High Sensitivity D5000 reagents (Cat. #5067-5593, Agilent) and further validated by single-cell qPCR of GAPDH or actin beta (ACTB) using the QuantiNova SYBR Green PCR Kit (Cat. #208052, Qiagen). The concentration was measured with the Quant-iT PicoGreen dsDNA Assay Kit (Cat. #P7581, Thermo Fisher Scientific) on the CLARIOstar Plate Reader (BMG Labtech, Ortenberg). Wells with Ct values for GAPDH or ACTB less than 20 were considered to contain high-quality cDNA. Good-quality cDNA was normalized to 2 μg/ml with EB buffer (Cat. #19086, Qiagen) into a new 96-well plate. Libraries were generated from the normalized single-cell cDNA using the miniaturized Nextera XT (Illumina) protocol [28] on a Mosquito HTS platform (SPT labtech), with 400 nl of input cDNA per reaction. The Nextera index sets A and D were used in parallel to multiplex cells. All libraries were cleaned up with a 0.6:1 ratio of Ampure XP beads (Cat. #A63881, Beckman Coulter). The multiplexed RNA-seq library was normalized to 4 nmol/l with Suspension Buffer (provided in the Nextera XT kit) and sequenced on a Hiseq 4000 Sequencer (Illumina).

### Bulk RNA-seq library preparation and sequencing

Total RNA of NCI-N87, MKN-74, SNU-719, and SNU-1 cancer cells was extracted using the RNeasy Mini Kit (Cat. #74104, Qiagen) according to the manufacturer’s instructions. RNA concentration was detected by the NanoDrop 2000 spectrophotometer (Thermo Fisher Scientific). After RNA quality was evaluated using the Fragment Analyzer (Agilent), mRNA was selected with the RNA NEBNext Poly(A) mRNA Magnetic Isolation Module (Cat. #E7490L, New England Biolabs, Inc.). Libraries were constructed using the NEBNext Ultra Directional RNA Library Prep Kit (Cat. #E7760S, New England Biolabs, Inc.). Sequencing was conducted on an Illumina NovaSeq 6000 sequencing platform, generating paired-end reads with a length of 150 bp over 150 cycles.

### Bulk RNA-seq data analysis

Quality control (QC) of RNA-seq reads was done by the Trim Galore pipeline (https://github.com/FelixKrueger/TrimGalore), which was a wrapper pipeline including the Cutadapt [29] and FastQC [30] tools. Reads that passed QC were mapped to the human genome (GRCh38) using STAR [31] with default parameters and filtered for uniquely mapped reads with SAMtools [32]. The number of reads per gene was counted with HTSeq (v0.11.3) [33]. Differentially expressed genes were identified using DESeq2 [34] with the criteria of |log_2_ fold change (FC)| > 1, and a false discovery rate (FDR) < 0.05. Transcripts per million mapped reads (TPM) were computed using a custom R script, which has been deposited on GitHub (https://github.com/tyxdavid/GastricCancerProject/blob/main/utils/Utils.R).

### Assay for transposase-accessible chromatin using sequencing library preparation and sequencing

NCI-N87 GC cells were dissociated from plates using trypsin (Cat. #25300-054, Gibco), and 2 × 10^5^ cells were collected and centrifuged at 350 ×*g* for 5 min. Cells were then washed with PBS, and centrifugation was repeated. Nuclei were centrifuged at 500 ×*g*, 4 °C for 10 min and resuspended in 10 μl of nuclease-free water (Cat. #AM9937, Thermo Fisher Scientific). The nuclei were then counted using a hemocytometer. Approximately 5 × 10^4^ nuclei were resuspended in 25 μl of Tagmentation buffer, 22.5 μl of nuclease-free water, and 2.5 μl of Tagmentation Enzyme from the Nextera DNA Library Prep Kit (Cat. #FC-131-1096, Illumina). The mixture was gently mixed and incubated in a 37 °C water bath for 30 min. The tagmentation reaction was stopped using MinElute PCR purification (Cat. #28004, Qiagen), and DNA was eluted in 10 μl of nuclease-free water. Assay for transposase-accessible chromatin using sequencing (ATAC-seq) library generation was performed using Illumina barcode oligos as described by Buenrostro et al. [35], with 8 to 11 cycles of PCR using NEBNext High Fidelity 2× PCR master mix (Cat. #M0541S, New England Biolabs, Inc.). The number of PCR cycles was empirically determined for each library by qPCR. Libraries were bioanalyzed using the High Sensitivity DNA Kit (Cat. #5067-4627, Agilent), pooled together, and sequenced on the Illumina NovaSeq 6000 platform using paired-end sequencing.

### ATAC-seq data analysis

Prealignment QC for FastQ files from ATAC-seq experiments was performed using the Trim Galore pipeline. Bowtie2 [36] was used to align reads to the GRCh38 genome with the parameters “--very-sensitive --no-discordant -X 2000”. Postalignment QC was conducted using SAMtools and Picard [37] to remove improperly paired reads, PCR duplicates, and reads mapped to mitochondrial genome or ENCODE blacklisted regions [38]. Peak calling was performed with MACS2 [39] using the parameters “-f BAMPE --shift -75 --extsize 150 --nomodel --call-summits --nolambda --keep-dup all”. To retain high-confidence peak regions, peaks identified from 2 biological replicate samples were intersected to obtain the overlapping peak regions.

### Cleavage under targets and tagmentation library preparation and sequencing

For cleavage under targets and tagmentation (Cut&Tag) sequencing, the experiment was conducted using the hyperactive universal Cut&Tag assay kit (Cat. #TD904, Vazyme) according to the manufacturer’s instructions. Briefly, approximately 5 × 10^4^ NCI-N87 cells were harvested, counted, and mixed with activated ConA Beads (included in the kit). The cells were incubated overnight with primary antibodies anti-CEBPB (Cat. #23431-1-AP, Proteintech), anti-H3K27ac (Cat. #13-0059, EpiCypher), and IgG negative control (Cat. #13-0047, EpiCypher). The anti-rabbit IgG secondary antibody (Cat. #Ab207, Vazyme) was incubated at room temperature with rotation for 1 h. After removing the supernatant, pA/G-Tnp Pro and Trueprep Tagment Buffer L were incubated separately with rotation at room temperature and 37 °C for 1 h. The supernatant was then mixed with DNA Extract Beads Pro (included in the kit) to extract DNA dissolved in ddH_2_O, which was directly used for PCR amplification. Purification of the amplified DNA was performed using VAHTS DNA Clean Beads (Cat. #N411-01, Vazyme). The purified DNA was subsequently subjected to next-generation sequencing on the Illumina NovaSeq 6000 platform.

### Cut&Tag data analysis

Prealignment QC for FastQ files from Cut&Tag experiments was performed using the Trim Galore pipeline. Bowtie2 was used to align reads to the GRCh38 genome with the parameters “--very-sensitive --no-unal --no-mixed --no-discordant -I 10 -X 700”. Postalignment QC was conducted using SAMtools and Picard to remove improperly paired reads, PCR duplicates, and reads mapped to the mitochondrial genome. Peak calling was performed using SEACR [40] with “nonstringent” parameters and IgG Cut&Tag sample data for background estimation. To retain high-confidence peak regions, peaks identified from 2 biological replicate samples were intersected to obtain the overlapping peak regions. IGV browser [41] was used for the visualization of ATAC-seq, H3K27ac Cut&Tag, and CEBPB Cut&Tag data.

### Identification of enhancers and their activation level

To identify genome-wide potential enhancers, we retained the overlapping regions of high-confidence peak regions from ATAC-seq and H3K27ac Cut&Tag and annotated their genomic locations using ChIPseeker [42]. A ±2,000-bp region flanking the transcription start site (TSS) was defined as the promoter region of the gene. The upstream region of the gene was defined as −100,000 bp to the TSS, and the downstream region was defined as +100,000 bp to the transcription end site (TES). Overlapping regions within gene transcripts (exons, introns, 3′ untranslated regions [UTRs], and 5′ UTRs), upstream regions, downstream regions, and intergenic regions were considered as potential enhancers. CEBPB-binding enhancers were defined as enhancer regions overlapping with at least one high-confidence peak from CEBPB Cut&Tag. Gene-level enhancer activation level was defined as the average H3K27ac modification level of potential enhancers that could be assigned to a specific gene (i.e., excluding intergenic enhancers). Only enhancers longer than 10 bp were included in this analysis.

To determine whether the enhancer of marker genes of each cell cluster or up-regulated genes of cell lines were more likely to be bound by CEBPB, enrichment analysis was conducted using single-sample gene set enrichment analysis (ssGSEA) [43] to evaluate the enrichment of the top 200 most active CEBPB-binding enhancer genes.

### Single-cell transcriptome library preparation and sequencing

All freshly sampled tissues were immediately rinsed with PBS after surgical resection. Each sample was then minced on ice into smaller pieces (<1 mm) and digested for 30 min at 37 °C with 300 ×*g* agitation in a digestion solution containing 0.2% collagenase I/II (Cat. #17018029, #17101015, Thermo Fisher Scientific), DNase I (Cat. #DN25, Sigma-Aldrich), and 25 units of dispase (Cat. #17105041, Thermo Fisher Scientific) in DMEM. The resulting cell suspension was filtered through 40-μm cell strainers (VWR) to remove aggregates and resuspended in DMEM supplemented with 10% FBS. Dead cells were eliminated by the membrane permeability dead cell apoptosis kit (Cat. #V13243, Thermo Fisher Scientific). A 10-μl aliquot of the cell suspension was counted using an automated cell counter (Luna) to determine the concentration of live cells.

scRNA-seq libraries were constructed using the Chromium Single Cell 3′ v3 workflow, Gel Bead & Multiplex Kit (Cat. #PN-1000121, 10x Genomics), and the Chip Kit (Cat. #PN-1000120, 10x Genomics), following the manufacturer’s instructions for recovering 4,000 cells. Briefly, the cell suspension was added to the master mix of the Gel Bead & Multiplex Kit containing nuclease-free water, reverse transcription (RT) reagent mix, RT primer, Additive A, and RT Enzyme Mix (included in the kit). The master mix with cells was transferred to the wells in row 1 of the Chromium Single Cell G Chip (10x Genomics). Single Cell 3′ Gel Beads were added to row 2, and partitioning oil was added to row 3. The chip was loaded onto the Chromium Controller to generate single-cell Gel Beads in Emulsion (GEMs). GEM-RT was performed in a C1000 Touch Thermal Cycler (Bio-Rad) under the following conditions: 53 °C for 45 min, 85 °C for 5 min, followed by a 4 °C hold. After GEM-RT, cleanup was performed using DynaBeads MyOne Silane Beads (Thermo Fisher Scientific). Complementary DNA (cDNA) was amplified using the C1000 Touch Thermal Cycler with the following cycling conditions: 98 °C for 3 min; 12 cycles of 90 °C for 15 s, 67 °C for 20 s, and 72 °C for 1 min; 72 °C for 1 min; followed by a 4 °C hold. Amplified cDNA was cleaned using the SPRIselect Reagent Kit (Cat. #B23318, Beckman Coulter), and quality was assessed with a 2100 Bioanalyzer (Agilent). All libraries were sequenced on an Illumina HiSeq 4000 platform with 150-bp paired-end reads.

### scRNA-seq data processing

FASTQ reads of 10x scRNA-seq data were aligned to the GRCh38 reference genome using Cell Ranger (v6.0) [44]. Publicly available scRNA-seq datasets GSE134520, GSE183904, and HRA000051 were downloaded from the GEO (www.ncbi.nlm.nih.gov/geo) and the GSA-human (ngdc.cncb.ac.cn/gsa-human) database, respectively. The processed expression matrices from different batches were merged, and subsequent analyses were performed using Seurat (v4.0) [45]. Cells with fewer than 500 unique molecular identifier (UMI) counts, fewer than 450 or more than 6,000 genes, or more than 50% of mitochondrial RNA counts were filtered out. Genes expressed in fewer than 3 cells were also removed. Possible remaining doublets were predicted for each sample using DoubletFinder [46], with an estimated doublet rate of 7.5%, and were excluded. Cells expressing known markers of 2 or more cell types after DoubletFinder prediction and exclusion were also manually removed. Notably, 395 putative hepatocytes from liver metastasis samples were also excluded due to dual expression of hepatocyte markers and T cell markers, including albumin (*ALB*), *CD45*, cell differentiation 3 delta chain (*CD3D*), and T cell receptor alpha chain constant region (*TRAC*). The filtered expression matrix was normalized by the total number of UMIs per cell and log_2_-transformed. Scaled data for all cells were used for principal component analysis (PCA) based on the top 2,000 highly variable genes. Harmony [47] was used to correct potential batch effect between patients, with the maximum number of iterations set to 50. The optimal harmony-corrected principal components for t-SNE embedding were determined using an elbow plot. The Leiden algorithm was then used to define clusters. Each main cell type/cluster was extracted and subjected to scaling, PCA, harmony, t-SNE, and Leiden clustering independently for further subcluster analysis.

Integrated analysis of the expression profiles from CTCs and PBMCs was also performed using the Seurat R package. The data were normalized using the TPM method, followed by scaling, PCA, and t-SNE visualization with Leiden clustering.

To rule out the possibility that the identified metastatic malignant cells represented resident hepatocytes within the liver, we performed an integrated scRNA-seq analysis. This involved comparing our identified malignant cells with reference hepatocytes obtained from the GSE115469 dataset [48].

### Tissue preference of each cell subcluster

To quantify the distribution preference of each cluster across tissues, we compared the observed and expected cell numbers (i.e., the ratio of observed to expected frequencies) in each subcluster according to the following formula, as described previously [49]:

$$R_{o/e}=\frac{\text{Observed}}{\text{Expected}}$$ (1)

where the expected cell numbers for each combination of cell subclusters and tissues were obtained from the chi-square test.

### The calculation of malignant scores by expression profiles

We calculated malignant and nonmalignant scores for epithelial cells using the AddModuleScore function in Seurat, with the malignant and the nonmalignant gene sets derived from a workflow adapted from Zhang et al. [16]. Only epithelial cells from metastases, primary tumors, and NATs were included. Briefly, the malignant and the nonmalignant gene sets for the first iteration were defined as the differentially expressed genes between tumor and normal tissue samples, determined using the Wilcoxon rank-sum test with an adjusted *P* (*P*_adj_) < 0.01 and |log_2_(FC)| > 1. Putative malignant and nonmalignant cells were then identified based on these scores using the K-means clustering algorithm (*K* = 2). In subsequent iterations, the malignant and the nonmalignant gene sets were redefined as the differentially expressed genes between putative malignant and nonmalignant epithelial cells, using the same threshold. Cells were reclassified based on the updated gene sets and scores, as described above. This process was repeated iteratively until the malignant and nonmalignant gene sets remained consistent with the previous iteration. Malignant cells identified from each epithelial subcluster were named using the prefix “Mag_” and a uniform suffix with the corresponding epithelial subcluster. For example, malignant cells identified from the Epi_FABP1 subcluster were designated as Mag_FABP1 hereafter.

### Copy number variation analysis

For each patient, epithelial cells from metastasis or primary tumor samples were considered as the putative tumor epithelium dataset, while epithelial cells from normal tissue samples were used as the reference dataset. The initial copy number variation (CNV) value of every single cell was estimated by inferCNV (v1.3.3) [50] based on transcriptomic profiles. To quantitatively evaluate the CNV level of each cell, we defined the CNV score as the mean square of CNV values across the genome.

### Gene set enrichment analysis and expression profile scoring

Gene set enrichment analysis (GSEA) and expression profile scoring were performed using ssGSEA with default parameters and then *z*-score transformed. The canonical pathway gene sets were downloaded from the MSigDB database [51].

### Organ-specific module expression analysis

Organ-specific modules, including liver modules, were defined as sets of highly expressed genes in each organ, identified with an FC > 1.5 and a *P*_adj_ < 0.01 compared to samples from other organs in the GTEx [52]. Organ-specific module expression scoring was performed using ssGSEA.

### Single-cell metabolic activity analysis

The computational pipeline for quantifying single-cell metabolism, scMetabolism (v0.2.1) [53], was applied. Specifically, the AUCell algorithm and metabolic pathways from the Reactome gene sets were used to quantify the metabolic activity of single cells in each cluster based on their expression profiles.

### Pseudotime analysis

We applied the single-cell trajectories analysis with Monocle2 [54] using the DDR-Tree algorithm and default parameters. The top 5,000 genes (ranked by *q*-value) that distinguished metastasis from primary tumor samples were used for cell ordering. Root state (the branch with the smallest pseudotime) was identified as the branch containing the highest number of nonmalignant cells. Dedifferentiation status was inferred using CytoTrace [55], and stemness score was calculated by the AddModuleScore function in Seurat using stemness marker genes for GC as described by Lin et al. [56]. To evaluate progression state similarity among cell clusters, the average Euclidean distance *D* between any 2 cell clusters, cluster A with *n_i_* cells and cluster B with *n_j_* cells, was calculated according to the following formula:

$$D_{\mathrm{AB}}=\frac{\sum_{y=1}^{j}\sum_{x=1}^{i}|p_{x}-p_{y}|}{n_{i}n_{j}}$$ (2)

where *p* denotes the inferred pseudotime of the cell.

### Expression profile similarity analysis between malignant cells and CTCs

Because the expression profiles of malignant cells and CTCs were profiled by distinctive sequencing methods, we sought to utilize the GSEA method to profile their similarity. The enrichment of the marker gene sets (defined as the top 50 highly expressed genes) of each malignant cell cluster was evaluated in the up-regulated genes of the 2 CTC clusters by the “GSEA” function in the clusterProfiler package (v4.8.1) [57]. A normalized enrichment score (NES) > 1 and a *P*_adj_ < 0.05 were considered indicative of significant enrichment, while higher NES indicated higher similarities.

### Cell–cell communication analysis

Cell–cell communication analysis was performed for all cell subclusters in metastasis and primary tumors separately, using CellChat (v1.6.1) [58]. Ligands and receptors expressed in more than 10 cells and in at least 5% of the total cells within a cluster were included in the analysis. Communication analysis was conducted with “population.size=TRUE”, taking the cell abundance into consideration. Communication events with a *P* < 0.05 were considered significant.

### Transcription factor analysis

To assess transcription factor (TF) activities, we performed the single-cell regulatory network inference and clustering (SCENIC) method [59] with the pySCENIC (v0.9.5) workflow using default parameters, and the RcisTarget database [59] was used to infer the regulatory relationship between TFs and their corresponding target genes.

### Microarray data analysis

For the public GC cohorts, the Asian Cancer Research Group (ACRG) cohort (GSE62254) and the Singapore (SG) cohort (GSE15459), gene expression of primary tumors was profiled using the Affymetrix HG-U133 Plus 2.0 array. Therefore, the Affy R package (v1.80.0) [60] was used for background correction (“rma”), normalization (“quantiles”), and probe set-level summarization (“medianpolish”). Probe–gene relationships were derived from the official annotation table (GEO database: GPL570). For genes with multiple corresponding probes, the probe with the highest variance was selected to represent the gene.

### Quantification and statistical analysis

All statistical analyses were conducted via the R software (version 4.1.0, https://www.r-project.org/). The Wilcoxon rank-sum test was employed to evaluate the significance of differentially expressed genes. For group comparisons, the independent-sample *t* test was used for 2 groups, and one-way analysis of variance (ANOVA) with Tukey’s test for post-hoc pairwise comparisons was applied for multiple groups. Kaplan–Meier survival analysis and the log-rank tests were conducted using the survival and survminer R packages. A 2-sided *P* < 0.05 was considered statistically significant.

## Results

### Single-cell RNA profiling identifies a heterogeneous cellular ecosystem in primary, CTCs, and metastatic lesions of GC

**Fig. 1.**
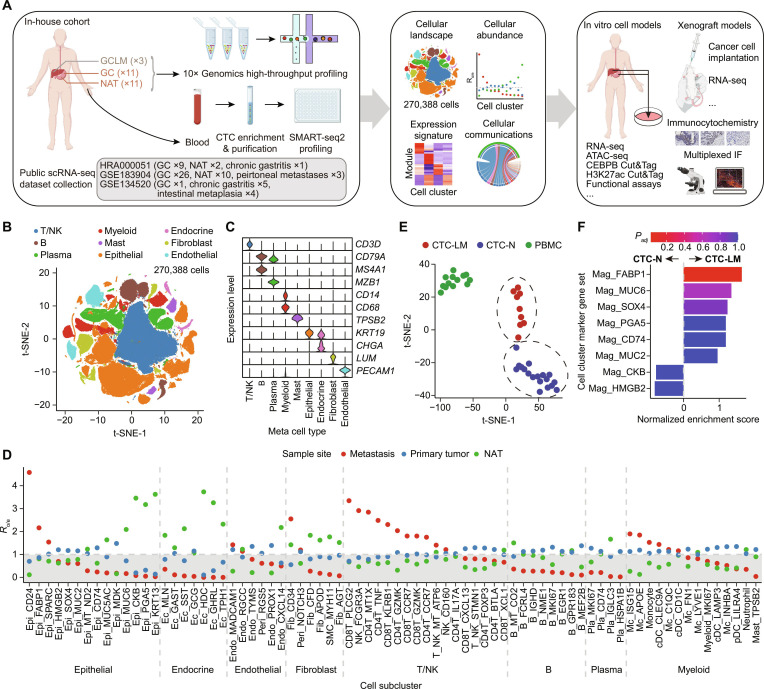
Single-cell RNA profiling identifies a heterogeneous cellular ecosystem in primary, CTC, and metastatic lesions of gastric cancer. (A) Overall study design delineating sample cohorts, analyses, and experimental validation methods. (B) t-SNE embedding and main cell-type assignment for each cell cluster. (C) Canonical marker expression level of each meta cell type. (D) Cell abundance of cell subclusters in metastasis, primary tumor, and NAT. (E) t-SNE embedding and clustering results for CTCs (*n* = 27) and PBMCs (*n* = 13). Two clusters of CTCs were identified. The cluster associated with liver metastasis was designated as CTC-LM, while the remaining cluster, not associated with liver metastasis, was annotated as CTC-N. (F) Expression profile similarities between the CTC clusters and the malignant cell clusters, evaluated using gene set enrichment analysis. Abbreviations: ATAC-seq, assay for transposase-accessible chromatin using sequencing; Cut&Tag, cleavage under targets and tagmentation; CTC, circulating tumor cell; CTC-LM, CTC associated with liver metastasis; CTC-N, CTC not associated with liver metastasis; IF, immunofluorescence; Endo, endothelial; Epi, epithelial; Fib, fibroblast; GC, gastric cancer; GCLM, gastric cancer liver metastasis; Mag, malignant; Mc, macrophage; NAT, normal adjacent tissue; PBMC, peripheral blood mononuclear cell; Peri, pericyte; Pla, plasma; *R*_o/e_, ratio of observed to expected frequencies; t-SNE, t-distributed stochastic neighbor; SMC, smooth muscle cell.

The expression profile of 11 primary tumors, 11 NATs, and 3 paired liver metastasis samples from 11 GC patients of the in-house cohort #1 was characterized at single-cell resolution by 10x Genomics technology (Fig. 1A and Table S1). To better portray a comprehensive cellular and molecular architecture of GC, we further integrated another 61 samples from 3 publicly available GC-related scRNA-seq datasets (HRA000051, GSE183904, and GSE134520), including 36 GC primary tumors, 3 peritoneal metastases samples, 12 NATs, 6 chronic gastritis tissues, and 4 intestinal metaplasia tissues. After QC and batch effect removal, a total of 270,388 cells from 59 patients were obtained (Fig. 1A and Fig. S1A). Based on expression of canonical cell-type-specific markers, we categorized these cells into 9 major cell clusters, including T/NK cells, B cells, plasma cells, myeloid cells, mast cells, epithelial cells, endocrine cells, fibroblasts, and endothelial cells (Fig. 1B and C). We next reclustered each major cluster to get subclusters with higher annotation resolution. Overall, 75 cell subpopulations were identified, including 14 epithelial, 7 endocrine, 6 endothelial, 6 fibroblast, 17 T/NK cell, 8 B cell, 4 plasma, and 14 myeloid cell subsets (Fig. S1B to D). We further recognized malignant cells from epithelial cells using the method proposed by Zhang et al. [16] and defined a malignant score and a nonmalignant score using distinguishable markers between malignant and nonmalignant cells (Fig. S2A). As expected, cells from metastasis samples had the highest malignant scores and the lowest nonmalignant scores, while cells from NATs showed opposite trends (Fig. S2B). The malignant cell classification result was further supported by inferCNV analysis, showing that malignant cells had higher CNV scores compared to nonmalignant cells (Fig. S2C). Malignant cells identified from each epithelial subcluster were named using the prefix “Mag_”, and a uniform suffix with the corresponding epithelial subcluster hereafter.

We next evaluated the metastatic preference of each cell subcluster (Fig. 1D). We found that epithelial cell clusters Epi_CD24, Epi_FABP1, and Epi_SPARC showed enriched distribution in metastasis niches. As expected, these metastasis-associated epithelial clusters showed the highest malignant scores compared to other epithelial clusters (Fig. S2D). Most of the T/NK cell subpopulations predominated in the metastasis sites, including CD8T_PLCG2, NK_FCGR3A, CD4T_MT1X, CD4T_TNF, CD8T_KLRB1, CD4T_GZMK, and CD8T_CCR7. The CD8T_KLRB1 cell cluster, a cluster of mucosal-associated invariant T (MAIT) cells highly expressing KLRB1, was thought to promote cancer initiation, growth, and metastasis in lung cancer [61]. CD8T_GZMK, characterized by high expression of granzyme K, has been reported to correlate with poor clinical outcomes in colorectal tumors [62]. In contrast, most B/plasma cell subpopulations were depleted at metastatic sites. For the myeloid cells, we found that macrophage cell clusters, such as Mc_ISG15 and Mc_APOE, were enriched in the metastasis sites (Fig. 1D). These cell clusters are known to play critical roles in tumor metastasis and immune suppression [63–65].

CTCs play a fundamental role in initiating tumor metastasis. To further track the culprit of metastasis traveling in circulation, we used a CTC sorting system, as previously described [66], to capture 27 CTC samples from 8 patients in our in-house cohort. These CTC samples were profiled by full-length scRNA-seq (SMART-seq2). We clustered CTC expression profiles along with the PBMCs and found that CTCs showed a distinct expression pattern compared with PBMC samples and had higher expression of keratin 9 (*KRT9*), mucin 5AC (*MUC5AC*), and mucin 6 (*MUC6*), in line with their gastric mucosa origins (Fig. S2E). Two clusters of CTCs were identified, and one of them included all 3 CTCs derived from patients with liver metastasis. We assumed that this CTC cluster might be associated with liver metastasis, and designated it as CTC-LM (CTC associated with liver metastasis), and the other CTC cluster as CTC-N (CTC not associated with liver metastasis; Fig. 1E). We next performed GSEA to assess the expression similarities between CTCs and malignant cell clusters derived from solid tissues. Interestingly, we found that CTC-LM showed the highest similarity with Mag_FABP1 (*P*_adj_ < 0.05 and NES > 1; Fig. 1F), indicating a close relationship between the metastasis-associated malignant cluster Mag_FABP1 and metastasis initiation.

### *CEBPB*-regulated cell plasticity is related to liver metastasis of GC cells

**Fig. 2.**
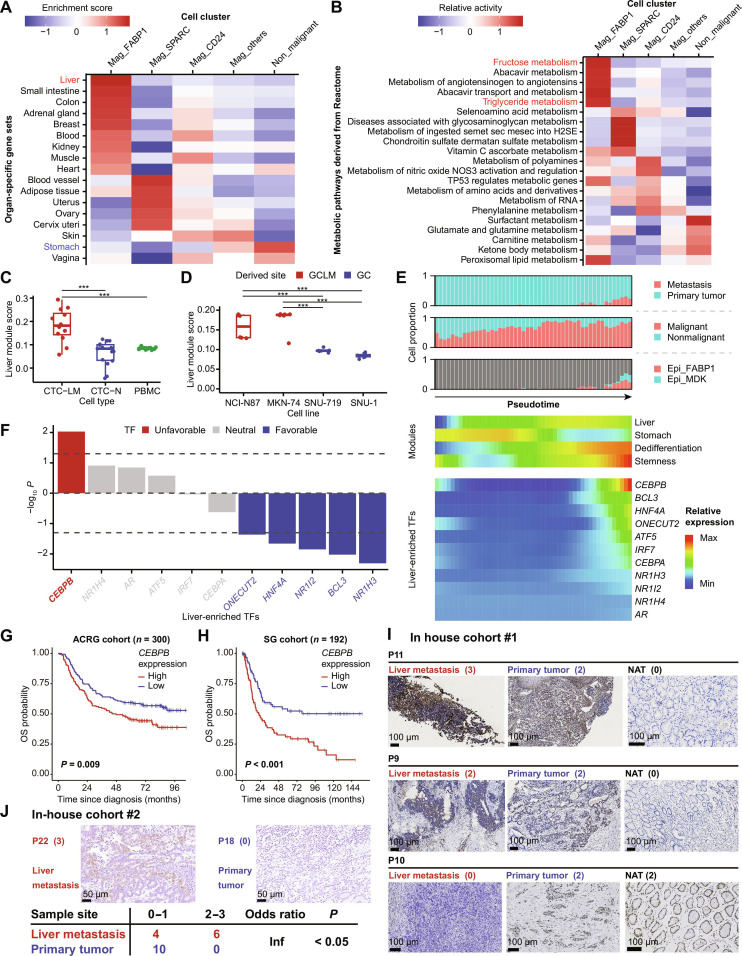
CEBPB-regulated gastric plasticity is associated with liver metastasis of GC cells. (A) Enrichment scores for organ-specific gene sets in malignant and nonmalignant cell clusters. (B) Relative activity of the top 5 metabolism pathways in malignant and nonmalignant cells inferred by scMetabolism. Liver-associated pathways are highlighted in red. (C) Liver module scores of CTC-LM, CTC-N, and PBMC. (D) Liver module scores of GC cell lines derived from GCLM (red) and primary GC tissues (blue). (E) Pseudotime analysis showing the proportion of cell subclusters (top), module scoring (middle), and the expression of liver metastasis-associated TFs (bottom) along the evolutionary trajectory. (F) Prognostic value of the liver metastasis-associated TFs shown in (E) in the ACRG cohort. *P* values were determined by log-rank test. (G and H) Kaplan–Meier plots showing the association of *CEBPB* expression with overall survival in the ACRG cohort (G) and SG cohort (H). Groups were stratified by the median *CEBPB* expression. *P* values were determined by log-rank test. (I) IHC staining of CEBPB in liver metastases, primary tumors, and NATs from 3 GC patients in in-house cohort #1. Overall intensity is indicated in the parentheses next to the patient ID. (J) IHC staining results of CEBPB in nonmatched liver metastasis and primary tumor samples from in-house cohort #2. Overall intensity is indicated in the parentheses next to the patient ID. Statistical significance was determined by Fisher’s exact test. Boxplots display the median value with whiskers extending to 1.5 times the interquartile range. Statistical significance, ***P < 0.001. Abbreviations: ATF5, activating transcription factor 5; AR, androgen receptor; BCL3, BCL3 transcription coactivator; CEBPA, CCAAT enhancer binding protein alpha; CEBPB, CCAAT enhancer binding protein beta; CTC, circulating tumor cell; CTC-LM, CTC associated with liver metastasis; CTC-N, CTC not associated with liver metastasis; Epi, epithelial; GC, gastric cancer; GCLM, gastric cancer liver metastasis; HNF4A, hepatocyte nuclear factor 4 alpha; IHC, immunohistochemistry; Inf, infinite; IRF7, interferon regulatory factor 7; Mag, malignant; NAT, normal adjacent tissue; NR1H3, nuclear receptor subfamily 1 group H member 3; NR1H4, nuclear receptor subfamily 1 group H member 4; NR1I2, nuclear receptor subfamily 1 group I member 2; ONECUT2, one cut homeobox 2; OS, overall survival; PBMC, peripheral blood mononuclear cell; TF, transcription factor.

We subsequently focused on characterizing the molecular features of Mag_FABP1 malignant cells to explore their potential mechanisms in initiating liver metastasis. By leveraging tissue-specific gene expression patterns derived from the GTEx dataset (Fig. S3A), we characterized the cellular identities of malignant cells. The Mag_FABP1 subtype exhibited a highly mixed cellular identity, with contributions from liver, intestine, colon, and kidney signatures, whereas nonmalignant cells maintained a predominantly gastric-specific gene expression pattern (Fig. 2A). This suggested that Mag_FABP1 cells are in a high plasticity cell state. Notably, the liver signature was most pronounced in the Mag_FABP1 subtype. Consistent with this identity, ssGSEA revealed that Mag_FABP1 cells had activated liver-associated processes, including chylomicron remodeling, cholesterol metabolism, and high-density lipoprotein remodeling (Fig. S3B), as well as fructose metabolism and triglyceride metabolism (Fig. 2B). To rule out the possibility that Mag_FABP1 represented a resident cluster of hepatocytes within the liver, we performed an integrated scRNA-seq analysis of Mag_FABP1 and known hepatocytes [48], and found distant marker gene expression patterns between them (Fig. S3C). These results indicate a comprehensive, transcriptionally driven reprogramming toward a liver-like metabolic profile. Moreover, we discovered that CTC-LM underwent a similar transition, as evidenced by a similar observation that CTC-LM exhibited a stronger hepatic-specific expression pattern compared to CTC-N (Fig. 2C). This phenomenon was also evident in vitro, as liver-metastatic GC cell lines, NCI-N87 and MKN-74, demonstrated an elevated liver-specific gene expression pattern compared to nonmetastatic GC cell lines, SNU-719 and SNU-1 (Fig. 2D). Collectively, these results suggested that a high plasticity state of GC malignant cells may play a critical role in the initiation of liver metastasis in GC.

We next sought to investigate how this plasticity was regulated in GC malignant cells during liver metastasis. Monocle 2 analysis revealed a tumor cell evolutionary trajectory associated with liver metastasis, where metastasis-enriched cells, including Epi_FABP1 cells, were located at the end of the pseudotime trajectory (Fig. 2E and Fig. S3D). Interestingly, we found that the hepatic-specific module score was up-regulated while the gastric-specific module score was down-regulated along the pseudotime trajectory (Fig. 2E), further demonstrating a liver-associated phenotypic switching during liver metastasis. Moreover, the dedifferentiation score inferred by CytoTRACE and stemness state were also enhanced along the trajectory (Fig. 2E), indicating the notion that cellular dedifferentiation orchestrates this phenotypic transition. SCENIC was performed to infer the potential regulatory network for each malignant cell cluster. This analysis revealed a list of TFs, including nuclear receptor subfamily 1 group I member 2 (*NR1I2*), one cut homeobox 2 (*ONECUT2*), CCAAT enhancer binding protein alpha (*CEBPA*), and *CEBPB*, which were highly active in Mag_FABP1 cells (Fig. S3E). Overlap analysis of these active TFs with the organ-specific up-regulated genes identified 11 liver-enriched TFs involved in phenotypic switching (Fig. S3F). Most of these TFs were up-regulated along the evolutionary trajectory, with CEBPB showing the most prominent increase (Fig. 2E). We then evaluated the association of these TFs with clinical outcomes of GC patients. *CEBPB* expression was shown to be the only risk factor in the ACRG cohort (Fig. 2F), in which high expression level of *CEBPB* was significantly associated with unfavorable overall survival (OS; Fig. 2G), and this association was further confirmed in another independent GC cohort, the SG cohort (Fig. 2H). The expression level of *CEBPB* was higher in the primary tumors of patients with liver metastasis compared to patients with other metastasis locations or no metastasis at all in the ACRG cohort (Fig. S3G). We further validated the expression levels of CEBPB in clinical samples from GC primary tumors, NATs, and liver metastasis by IHC staining. In cohort #1 with matched liver metastasis, primary tumor, and NATs from 3 patients, CEBPB showed the highest expression level in liver metastasis, moderate expression in primary tumors, and almost no expression in NATs in 2 patients (Fig. 2I). To account for the interpatient heterogeneity observed in cohort #1, we further performed IHC on cohort #2, which consisted of unpaired primary tumors and liver metastases collected from 20 independent patients. Consistently, we found enriched expression of CEBPB in liver metastases compared to primary tumors (Fig. 2J). In addition, liver-metastatic GC cell lines, NCI-N87 and MKN-74, had significantly higher CEBPB expression compared to nonmetastatic cell lines, SNU-1 and SNU-719 (Fig. S3H and I). These results suggested that *CEBPB* had the potential to mediate liver-associated cell plasticity in GC cells during liver metastasis.

Another interesting finding was that a primary tumor-enriched malignant cell cluster, Mag_MDK, also showed an elevated hepatic-specific module score (Fig. S3J) and elevated expression levels of *CEBPB* (Fig. S3K). Epi_MDK had the closest relationship with Epi_FABP1 compared to other epithelial cell clusters (Fig. S3L and M), and had a similar evolutionary fate with Epi_FABP1 (Fig. 2E and Fig. S3D). These suggested that liver-associated phenotypic switching may initiate in the primary tumors.

### *CEBPB* regulates cell plasticity through its global enhancer-binding function

**Fig. 3.**
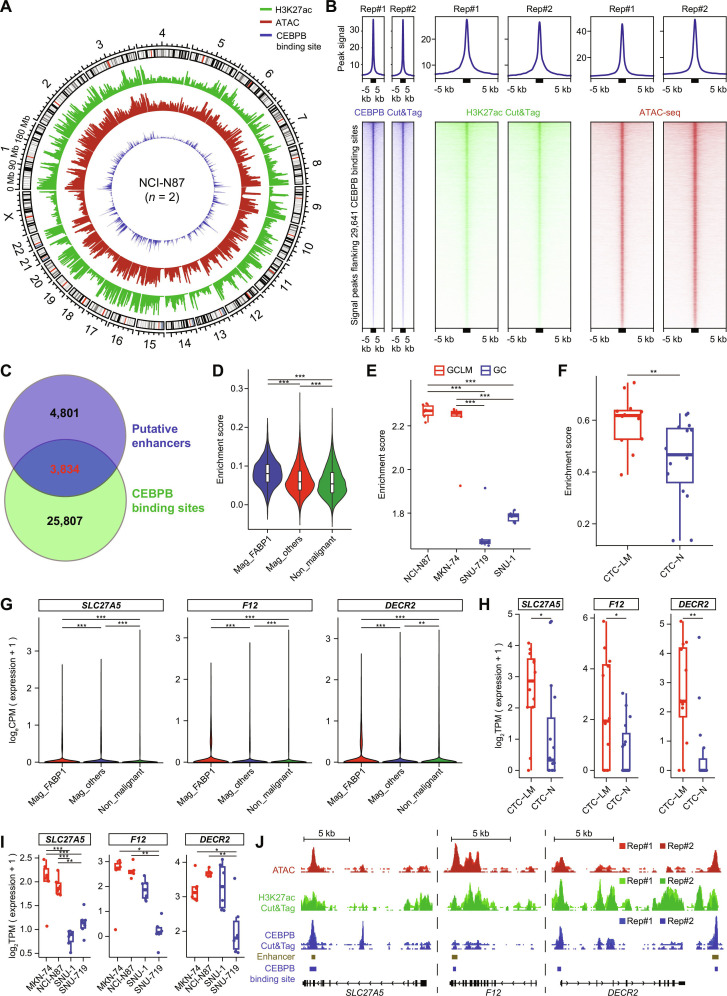
CEBPB regulates gastric plasticity through its global enhancer-binding function. (A) H3K27ac modification levels, chromosomal accessibility (defined by ATAC-seq signals), and the number of CEBPB-binding sites across the genome in the NCI-N87 (*n* = 2). (B) CEBPB Cut&Tag, H3K27ac Cut&Tag, and ATAC-seq peak signal levels flanking ±5 kb of the CEBPB binding sites (indicated by the black box) in NCI-N87 (*n* = 2). (C) Venn diagrams showing the overlap between putative enhancers and CEBPB binding sites. (D) Gene set enrichment analysis of malignant and nonmalignant cell clusters using the top 200 most active CEBPB-binding enhancer genes derived from NCI-N87 H3K27ac Cut&Tag data. (E) Gene set enrichment analysis of transcriptome profiles of GC cell lines derived from GCLM (red) and primary GC tissues (blue) using the top 200 most active CEBPB-binding enhancer genes derived from NCI-N87 H3K27ac Cut&Tag data. (F) Gene set enrichment analysis of transcriptome profiles of CTCs using the top 200 most active CEBPB-binding enhancer genes derived from NCI-N87 H3K27ac Cut&Tag data. (G to I) Expression levels of liver-specific genes (*SLC27A5*, *F12*, and *DECR2*) in malignant and nonmalignant cells (G), CTCs (H), and GC cell lines (I). (J) ATAC-seq, H3K27ac Cut&Tag, and CEBPB Cut&Tag signal levels, along with putative enhancer and CEBPB binding regions, in the gene regions of *SLC27A5*, *F12*, and *DECR2* in NCI-N87 (*n* = 2). Statistical significance, *P < 0.05, **P < 0.01, ***P < 0.001. Abbreviations: ATAC-seq, assay for transposase-accessible chromatin using sequencing; CEBPB, CCAAT enhancer-binding protein beta; CPM, counts per million; Cut&Tag, cleavage under targets and tagmentation; CTC, circulating tumor cell; CTC-LM, CTC associated with liver metastasis; CTC-N, CTC not associated with liver metastasis; DECR2, 2,4-dienoyl-CoA reductase 2; F12, coagulation factor XII; GC, gastric cancer; GCLM, gastric cancer liver metastasis; Mag, malignant; Rep, replicate; SLC27A5, solute carrier family 27 member 5; TPM, transcripts per million.

It is known that enhancer plays a critical role in regulating cell plasticity and *CEBPB* is an enhancer-binding transcriptional factor [67–70]. We therefore performed ATAC-seq, H3K27ac Cut&Tag sequencing, and CEBPB Cut&Tag sequencing in liver-metastatic NCI-N87 to investigate the mechanism by which *CEBPB* regulated cell plasticity (Fig. 3A). We found that the genomic regions around the CEBPB-binding sites revealed by CEBPB Cut&Tag sequencing were substantially enriched with ATAC-seq signals and H3K27ac Cut&Tag sequencing signals (Fig. 3B), indicating a global enhancer-binding function of *CEBPB*. The genomic sites distal to TSSs and with both H3K27ac and ATAC-seq signals were defined as potential active enhancers, which revealed 8,635 putative active enhancers. Among these active enhancers, 3,834 were also bound by CEBPB (Fig. 3C). Interestingly, we found that CEBPB-binding enhancers were significantly activated in tumor cells associated with liver metastasis, as demonstrated by the data from scRNA-seq (Fig. 3D), GC cell lines (Fig. 3E), and CTCs (Fig. 3F). Moreover, we found that a proportion of (173, 9.47%) liver-specific genes contained CEBPB-binding enhancers (Table S3). For example, liver-specific genes including solute carrier family 27 member 5 (*SLC27A5*), coagulation factor XII (*F12*), and 2,4-dienoyl-CoA reductase 2 (*DECR2*) were up-regulated in tumor cells associated with liver metastasis (Fig. 3G to I and Fig. S3N). These genes contained enhancers bound by CEBPB, indicating that their expression was up-regulated upon CEBPB binding (Fig. 3J). Taken together, CEBPB can regulate cell plasticity during liver metastasis through its global enhancer-binding function.

### *CEBPB* promotes liver metastasis of GC

We next thought to interrogate the impact of *CEBPB*-mediated cell plasticity on the biological behaviors of GC cells. Through calculating the invasion and migration score using ssGSEA, we found that Mag_FABP1 and liver-metastatic GC cell NCI-N87 and MKN-74 had significantly higher invasion and migration scores (Fig. S4A and B). Moreover, samples with high expression levels of *CEBPB* showed significantly higher invasion and migration scores in the 2 public cohorts (Fig. S4C and D). These findings provided preliminary hints of pro-malignancy and pro-metastasis ability of *CEBPB*. Based on the aforementioned expression status of *CEBPB* in GC cell lines (Fig. S3H), we knocked down the expression of *CEBPB* in NCI-N87 and MKN-74 by shRNAs (Fig. S4E) and overexpressed *CEBPB* in SNU-1 and SNU-719 (Fig. S4F) to investigate the role of *CEBPB* in pro-metastasis in vitro. The results showed that sh*CEBPB* cell lines had significantly reduced proliferation, invasion, and migration abilities compared to the corresponding control cell lines (Fig. S4G to I), while CEBPB-OE cell lines exhibited enhanced proliferation, invasion, and migration abilities compared to the control cell lines (Fig. S4J to L).

**Fig. 4.**
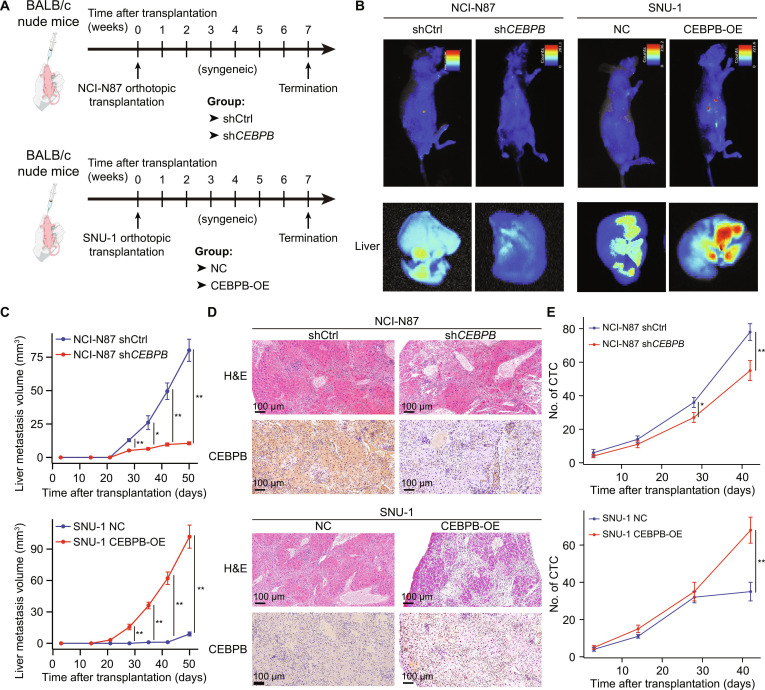
CEBPB enhances liver colonization and metastasis of GC cells in vivo. (A) Scheme of in vivo mouse experiments (*n* = 5 for each group). (B) Cancer metastasis status was visualized using an in vivo bioluminescence imaging system on day 50. (C) Tumor volume of liver metastases inferred from in vivo bioluminescence imaging results performed every 7 days from day 3 to day 50 since cell transplantation. (D) Representative H&E staining and IHC results for CEBPB in liver metastasis tissues from the NCI-N87 sh*CEBPB* group, SNU-1 CEBPB-OE group, and their corresponding control group. The imaging procedures were performed on all biological replicates per group. (E) Captured number of CTCs in the NCI-N87 sh*CEBPB* group, SNU-1 CEBPB-OE group, and their corresponding control group. Data were presented as the mean ± standard deviation. Statistical significance, *P < 0.05, **P < 0.01. Abbreviations: CEBPB, CCAAT enhancer-binding protein beta; CTC, circulating tumor cell; GC, gastric cancer; H&E, hematoxylin and eosin; IHC, immunohistochemistry; OE, overexpression; NC, negative control; shCEBPB, CEBPB knockdown by shRNA.

The functional role of *CEBPB* in GC liver metastasis was then evaluated in mouse tumor xenografts, in which the BALB/c nude mice were treated with intragastric injection (injected under the gastric serosal layer) implantation (Fig. 4A). It was found that mice implanted with sh*CEBPB* cells presented weak luciferase activity in the liver and smaller tumor size, indicating impaired liver metastasis ability, while mice implanted with CEBPB-OE cells showed stronger luciferase activity in the liver and larger tumor size, suggesting enhanced liver metastasis ability (Fig. 4B and C), which was further validated by histological evaluation (Fig. 4D). We also monitored the dynamic changes in the number of CTCs in these mouse xenograft models to track liver metastasis dynamics in the blood circulation. Compared with the corresponding control cell lines, detected CTCs showed reduced frequency in xenografts transplanted with sh*CEBPB* cells but increased frequency in xenografts transplanted with CEBPB-OE cells (Fig. 4E). Collectively, these in vitro and in vivo observations suggested the positive regulatory roles of *CEBPB* in mediating the metastasis-related capability of GC.

### *CEBPB*-regulated cell plasticity evades CD8^+^ T cell surveillance by CD155–TIGIT interaction

**Fig. 5.**
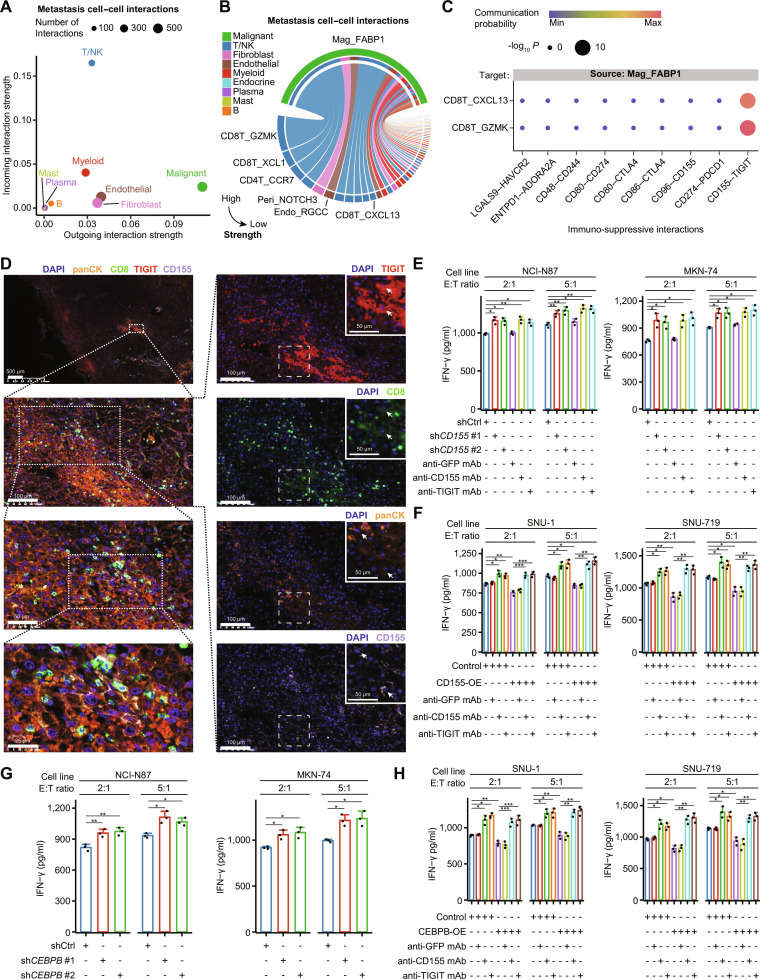
CEBPB-regulated gastric plasticity evades CD8^+^ T cell surveillance by CD155–TIGIT interaction in vitro. (A) Overall incoming and outgoing interaction strength of each meta-cell cluster. (B) Overall interaction strength between Mag_FABP1 and other tumor microenvironment cell subclusters in metastases. (C) Communication probability of canonical immuno-suppressive interactions between Mag_FABP1 and exhausted CD8T_CXCL13 or pre-exhausted CD8T_GZMK in metastases. (D) Multiplex immunofluorescent staining of a GC liver metastasis sample. The slide was stained with a multiplex quantitative immunofluorescence panel including DAPI (blue), cytokeratin (panCK, orange), CD8 (green), TIGIT (red), and CD155 (purple). A zoomed-in picture is shown in better resolution at the upper right corner, and the example TIGIT^+^CD8^+^ T cells and CK^+^CD155^+^ cancer cells were indicated by white arrows. (E and F) Levels of IFN-γ secreted by CD8^+^ T cells cocultured with liver-metastatic GC cells (E) or nonmetastatic GC cells (F) under different treatments. (G and H) Levels of IFN-γ secreted by CD8^+^ T cells cocultured with sh*CEBPB* liver-metastatic GC cells (G) or CEBPB-OE nonmetastatic GC cells under different treatments (H). Data were presented as the mean ± standard deviation. Statistical significance, *P < 0.05, **P < 0.01, ***P < 0.001. Abbreviations: ADORA2A, adenosine A2a receptor; CD244, cluster of differentiation 244; CD274, cluster of differentiation 274; CD48, cluster of differentiation 48; CD8, cluster of differentiation 8; CD80, cluster of differentiation 80; CD86, cluster of differentiation 86; CD96, cluster of differentiation 96; CD155, cluster of differentiation 155; CEBPB, CCAAT enhancer binding protein beta; CK, cytokeratin; CTLA4, cytotoxic T-lymphocyte associated protein 4; Endo, endothelial; ENTPD1, ectonucleoside triphosphate diphosphohydrolase 1; E:T ratio, effector:target ratio; GC, gastric cancer; mAb, monoclonal antibody; GFP, green fluorescent protein; HAVCR2, hepatitis A virus cellular receptor 2; IFN-γ, interferon-gamma; LGALS9, galectin 9; OE, overexpression; Mag, malignant; mAb, monoclonal antibody; PDCD1, programmed cell death 1; Peri, pericyte; shCD155, CD155 knockdown by shRNA; shCEBPB, CEBPB knockdown by shRNA; TIGIT, T cell immunoreceptor with Ig and ITIM domains.

To decipher how CEBPB-mediated cell plasticity facilitates adaptation to the liver microenvironment in GC, we focused on its ability to promote immune evasion. CellChat analysis showed that the malignant cell cluster was the major signaling sender of cellular communication, while the T/NK meta cell cluster was the major signaling receiver in the metastatic site (Fig. 5A). Further analysis of T cells from metastases samples revealed 2 exhausted CD8^+^ T cell clusters, CD8T_CXCL13 and CD8T_GZMK, which expressed multiple markers indicative of exhaustion, including cytotoxic T-lymphocyte associated protein 4 (*CTLA4*), programmed cell death 1 (*PDCD1*), layilin (*LAYN*), lymphocyte activating 3 (*LAG3*), hepatitis A virus cellular receptor 2 (*HAVCR2*), and *TIGIT* [71] (Fig. S5A). We found that Mag_FABP1 had strong interactions with the CD8T_GZMK clusters and moderate interactions with the CD8T_CXCL13 clusters (Fig. 5B and Fig. S5B). Mag_FABP1 interacted with CD8T_GZMK and CD8T_CXCL13 cells through the CD155–TIGIT ligand–receptor pair in the liver-metastatic site (Fig. 5C).

The interaction between cancer cells and CD8^+^ T cells via the CD155–TIGIT pair was validated in liver metastasis samples of GC patients by multiplex immunofluorescence staining, which showed colocalization of CD8^+^ T cells (CD8) and cancer cells (cyto-keratins and panCK), along with the interacting CD155–TIGIT ligand–receptor pair (Fig. 5D). We then cocultured GC cells with CD8^+^ T cells in vitro to confirm the immunosuppressive role of CD155–TIGIT interaction. We found that CD8^+^ T cells had significantly enhanced cytotoxicity ability with elevated production of IFN-γ and perforin when cocultured with sh*CD155* NCI-N87 or MKN-74 cells, and the introduction of anti-CD155 mAbs or anti-TIGIT mAbs into the cocultured assays showed a similar effect (Fig. 5E and Fig. S6A and B). Compared with corresponding controls, CD8^+^ T cells displayed significantly reduced cytotoxicity when cocultured with CD155-OE SNU-1 or SNU-719 cells, and such an effect could be reversed by adding anti-CD155 mAbs or anti-TIGIT mAbs into the cocultured system (Fig. 5F and Fig. S6A and C). We further verified whether *CEBPB* could promote cancer cell immune evasion through CD155–TIGIT interaction. *CEBPB* expression was shown to positively regulate *CD155* expression (Fig. S6D), which was further supported in the ACRG and SG cohort (Fig. S6E). Additionally, ATAC-seq, H3K27ac Cut&Tag, and CEBPB Cut&Tag of NCI-N87 GC cells suggested that a CEBPB-binding enhancer was located in the 3′ UTR region of *CD155* (Fig. S6F), which was further validated by the luciferase reporter assays (Fig. S6G). Compared with the corresponding controls, CD8^+^ T cells showed enhanced cytotoxicity when cocultured with sh*CEBPB* NCI-N87 and MKN-74 cells (Fig. 5G and Fig. S6H) but impaired cytotoxicity when cocultured with CEBPB-OE SNU-1 and SNU-719 cells (Fig. 5H and Fig. S6I). Moreover, reduced CD8^+^ T cell cytotoxicity in the CEBPB-OE coculture system could be reversed when introducing anti-CD155 or anti-TIGIT mAbs (Fig. 5H and Fig. S6I).

**Fig. 6.**
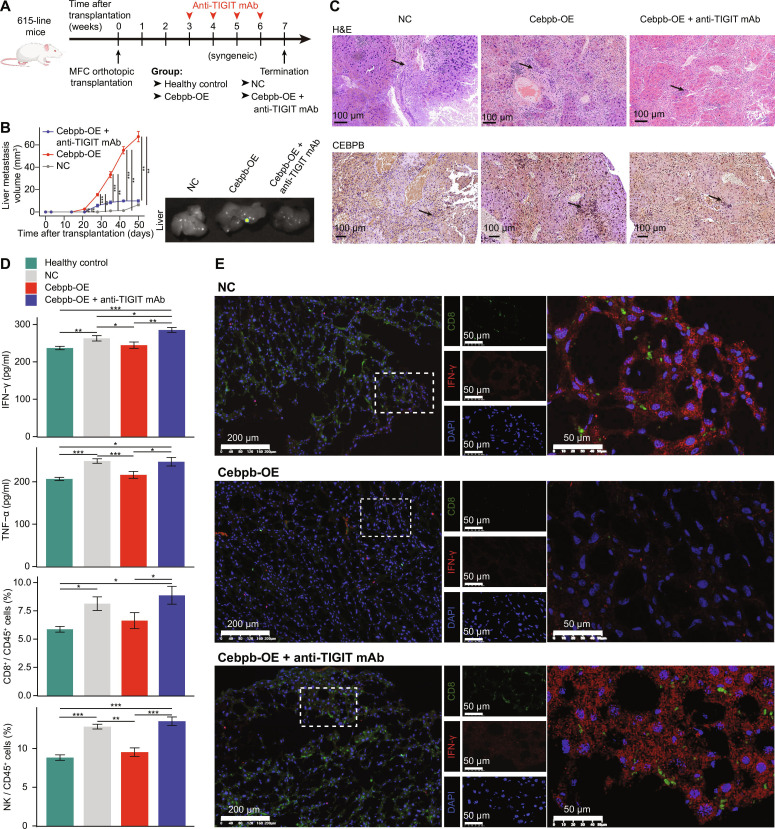
Cebpb-regulated gastric plasticity evades CD8^+^ T cell surveillance by CD155–TIGIT interaction in vivo. (A) Schematic of in vivo mouse experiments to determine the immune-regulatory role of Cebpb (*n* = 5 for each group). The healthy control group denoted the group without cancer cell transplantation, and the NC group denoted the group transplanted with MFC. (B) Tumor volume of liver metastases inferred from in vivo bioluminescence imaging results performed every 7 days from day 3 to day 50 (left) since cell transplantation, and liver metastases visualized using a bioluminescence imaging system on day 50 (right). (C) Representative H&E staining and immunohistochemistry results for Cebpb in liver metastasis tissues from the control group, MFC Cebpb-OE group, and Cebpb-OE plus anti-TIGIT mAbs treatment group. Arrows denote the representative areas of GC liver metastasis within the hepatic tissue. (D) Levels of IFN-γ, TNF-α, and the proportion of CD8^+^ T cells and NK cells among CD45^+^ immune cells in the healthy control group (green), MFC control group (gray), Cebpb-OE group (red), and Cebpb-OE plus anti-TIGIT mAbs treatment group (blue). (E) Multiplex immunofluorescence showing CD8 (green), IFN-γ (red), and nuclei (blue) in the MFC control group, Cebpb-OE group, and Cebpb-OE plus anti-TIGIT mAbs treatment group. Data were presented as the mean ± standard deviation. Statistical significance, *P < 0.05, **P < 0.01, ***P < 0.001. Abbreviations: Cebpb, CCAAT enhancer binding protein beta; H&E, hematoxylin and eosin; IFN-γ, interferon-gamma; mAb, monoclonal antibody; NC, negative control; OE, overexpression; TNF-α, tumor necrosis factor alpha; TIGIT, T cell immunoreceptor with Ig and ITIM domains.

The regulatory relationship between *Cebpb* and CD155–TIGIT was further evaluated in mouse models. The mouse GC cell line, MFC, and its immune-competent host, mouse strain 615, were utilized for evaluation [72] (Fig. 6A and Fig. S7). Mice implanted with Cebpb-OE MFC showed more progressive liver metastasis, while the application of anti-TIGIT mAb could reverse such effect (Fig. 6B and C). Decreased levels of IFN-γ and TNF-α, as well as reduced proportion of CD8^+^ T cells and NK cells, were observed in serum samples from mouse model transplanted with Cebpb-OE MFC, while such an effect could be reversed by anti-TIGIT mAbs (Fig. 6D). Multiplex immunofluorescence staining further validated not only the reduced expression of IFN-γ by CD8^+^ T cells in the mouse model transplanted with Cebpb-OE MFC, but also the reverse effect of anti-TIGIT mAbs (Fig. 6E). Altogether, our findings demonstrated that *CEBPB* positively regulated *CD155* expression and helped reprogrammed GC cells evade immune surveillance of CD8^+^ T cells.

## Discussion

Liver metastasis, as the most frequent distant disseminated organ in GC, is challenged by a grim quality of life and limited therapeutic efficacy. In the present study, we integrated single-cell transcriptome, bulk transcriptome, epigenomic resources, and experimental models to investigate how the culprit of metastasis spreads to and adapts to the new liver microenvironment. We constructed a comprehensive transcriptome atlas of GC primary tumors, NATs, paired liver metastasis, and CTCs at a single-cell scale, which revealed substantial intratumoral and intertumoral heterogeneity. We found that liver-metastatic GC cells were transcriptionally reprogrammed to gain liver-associated expression patterns, namely, a liver-associated phenotypic switching, possibly for better nutrient uptake and utilization in a distinct microenvironment [73–75].

Several studies have emphasized the important roles of organ-specific reprogramming for cancer cells in organ-specific metastasis (“mimicry”), including bone, brain, and lung metastasis [76–79], suggesting the significance of reprogramming for cancer cells in adapting to and surviving in the microenvironment of the distant metastatic sites [80], in line with the “seed and soil” hypothesis [81]. Whether liver-associated transcriptional reprogramming in GC represents a common behavior that also occurs in and promotes liver metastasis of other cancers, and whether differences and commonalities among them are associated with disease progression, warrants further investigation. To dissect how these reprogrammed cancer cells adapt to metastatic sites, we conducted cell–cell interaction analysis to reveal the cellular crosstalk within TME and discovered that the reprogrammed cancer cells actively interacted with pre-exhausted and exhausted CD8^+^ T cells. The immunosuppressive interactions were then explored, and the CD155–TIGIT interaction was uncovered and further validated in vitro and in vivo. Notably, given that the MFC cell line used for our in vivo experiments originates from the mouse forestomach (squamous-type epithelium), it is plausible that the CEBPB-CD155–TIGIT regulatory axis orchestrates a broader program of phenotypic switching that extends beyond glandular stomach adenocarcinoma to include squamous cell carcinomas. Acknowledging this potential generalizability highlights the broad relevance of the CEBPB-driven metastatic program in epithelial cancer progression. Though CD155–TIGIT interaction has been shown to induce T cell exhaustion in primary tumors of GC [82], our work further extends our understanding of its involvement in liver metastasis. In addition, we found no PDCD1–cluster of differentiation 274 (CD274) interaction between these cancer cells and CD8^+^ T cells, which might, to some extent, explain the limited response to programmed death-1 (PD-1)/programmed death-ligand 1 (PD-L1) immune checkpoint inhibitors in liver-metastatic GC patients [83]. This necessitates the application of alternative immune checkpoint inhibitors, such as CD155/TIGIT inhibitors, in advanced GC. Clinical trials investigating TIGIT blockade as a monotherapy or in combination with PD-1/PD-L1 blockade are ongoing at various stages and are highly anticipated [84,85].

Dysregulated TF activities mediate aberrant gene expression, which can be found in various types of cancer [86]. We uncovered a panel of liver-associated TFs that showed up-regulation during GC cell transition, in which *CEBPB* showed the most salient association and correlated with poor prognosis in GC. Coordinating with other liver-associated TFs [87], like *CEBPA*, *CEBPB* is known as a key regulator for liver development [69] and hepatocyte lineage differentiation [88,89], based on which we speculated that it possessed the ability to enable liver-associated transcriptional reprogramming. Indeed, despite the interpatient heterogeneity exemplified by Patient #10, we observed up-regulated expression of *CEBPB* in liver metastasis compared to primary tumors and NATs, indicating potential roles of *CEBPB* in liver-associated reprogramming and metastasis of GC. Such variability, as observed in Patient #10, likely reflects the complex clinical reality of GCLM, where individual differences in the tumor immune microenvironment, or the clonal evolution of metastatic seeds, may modulate TF activity. Acknowledging this heterogeneity not only strengthens the credibility of our dataset but also emphasizes the potential necessity for personalized therapeutic strategies when targeting the CEBPB-driven metastatic axis. Further experimental models validated that *CEBPB* could promote the malignant biological behaviors of GC. In addition, the roles of *CEBPB* in promoting cancer progression in breast cancer, ovarian cancer, and colorectal cancer have also been recorded [90–92], highlighting the prospect of targeting CEBPB for cancer treatment in various cancers. Preliminary studies have delineated the antitumor activities of some small-molecule CEBPB inhibitors, including betulinic acid, which can directly block the DNA binding of CEBPB [93], and withaferin A, which interferes with protein–protein interactions of CEBPB [94]. However, directly targeting CEBPB remains challenging due to difficulties in dealing with the complex molecular conformation of protein–DNA binding and protein–protein interactions [86]. The inhibition of upstream regulators and downstream effectors of the *CEBPB* regulatory axis may serve as an alternative and indirect approach for CEBPB interference. For example, silencing signal transducer and activator of transcription 3 (*STAT3*) may suppress *CEBPB* expression [95,96]. We further investigated the regulatory mechanism of *CEBPB* and found its role in reshaping the landscape of enhancer activation. In particular, liver-specific expression genes contributing to reprogramming were regulated by CEBPB via enhancer activation. The alteration of enhancer activation is common in tumorigenesis and has been proposed as a driving event of transcriptional reprogramming [97]. Therefore, inhibition of enhancer activity has potential to treat tumors, and the coadministration of CEBPB inhibitor and enhancer inhibitor may be an effective and feasible approach.

**Fig. 7.**
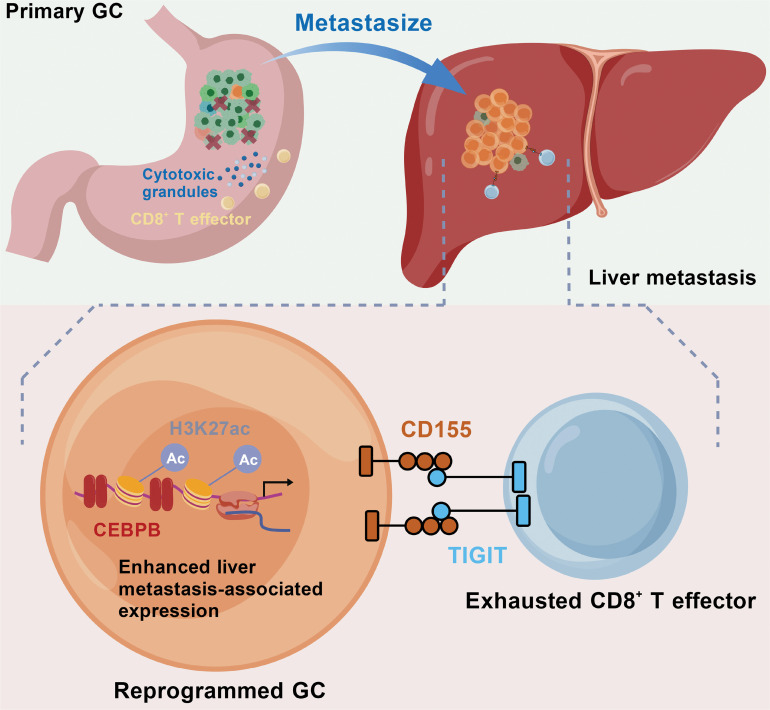
A conceptual model illustrating CEBPB-regulated gastric cell plasticity that promotes liver metastasis of GC. CEBPB-driven transcriptional and enhancer reprogramming confers high plasticity to GC cells, thereby activating liver metastasis-associated genes and promoting liver metastasis by inducing CD8^+^ T cell exhaustion via the CD155–TIGIT interaction. Abbreviations: Ac, acetylation; CD155, cluster of differentiation 155; CEBPB, CCAAT enhancer binding protein beta; GC, gastric cancer; TIGIT, T cell immunoreceptor with Ig and ITIM domains.

In summary, our work provided insights into the cellular and molecular mechanisms of liver metastasis of GC. Liver-metastatic GC cells acquired phenotypic switching via *CEBPB*-mediated liver-associated transcriptional reprogramming, and evaded immune surveillance from exhausted CD8^+^ T cells through CD155–TIGIT interaction in the liver-metastatic niche (Fig. 7). Our findings hold profound significance in the understanding of cancer development and in the development of effective treatment strategies to fight against GC liver metastasis.

This study has several limitations. Our scRNA-seq analysis has revealed that the *CEBPB*^+^ malignant cell cluster, Mag_MDK, may be the source of Mag_FABP1, further suggesting the initiation of liver-associated phenotype switching in primary tumor sites, but we were unable to further validate this assumption. Future work employing single-cell DNA sequencing and lineage tracing will be essential to delineate the origin of highly plastic GC cells during metastasis. Furthermore, while our epigenomic analysis identified multiple CEBPB-regulated putative enhancers and we functionally validated one controlling CD155 expression via luciferase reporter assays (in vitro), the in vivo relevance of these regulatory elements warrants further investigation. Cutting-edge technologies, such as the enCRISPRa and enCRISPRi systems [98], could be utilized in xenograft models to achieve precise in vivo functional validation of these enhancers.

## Ethical Approval

This study was approved by the Ethics Committee of Zhongshan Hospital of Fudan University (B2023-289R) and the Ethics Committee of Medical Discovery Leader Co., Ltd. (MDL2023-08-28-01).

## Data Availability

The raw sequencing data for this study have been deposited at the National Genomics Data Center (NGDC, https://ngdc.cncb.ac.cn/gsa-human) with accession number: HRA007757. The code supporting this study has been deposited at GitHub (https://github.com/tyxdavid/GastricCancerProject). Additional datasets used for this study were obtained from public repositories, including 4 scRNA-seq datasets curated from the GEO database (GSE183904, Kumar et al., 2022 [14]; GSE134520, Zhang et al., 2019 [15]; and GSE115469, MacParland et al., 2018 [48]) and the GSA database (HRA000051, Zhang et al., 2021 [16]), a PBMC RNA-seq dataset from the GEO database (GSE107011, Monaco et al., 2019 [100]), and transcriptomic profiles of GC patients from the ACRG cohort (GSE62254, Cristescu et al., 2015 [101]) and the SG cohort (GSE15459, Ooi et al., 2009 [102]).
