# Supplementary material for: CEBPB-Regulated Gastric Cell Plasticity Promotes Liver Metastasis of Gastric Cancer
Source: Cancer Commun (Lond). 2026 Mar 12;46:0016. doi: 10.34133/cancomm.0016 (PMC12981248; doi:10.34133/cancomm.0016)
Supplement: Supplementary 1 — Figs. S1 to S7 Tables S1 to S3 [file cancomm.0016.f1.pdf]

Supplementary Materials for

CEBPB-regulated gastric cell plasticity promotes liver metastasis of gastric cancer

Zhixiang Zuo[1,†,*], Jiaqi Liang[1,†], Li-Na He[1,2, †], Kai Xu[3,†], Muren Hu[4,†], Kunming Zhang[5], Wei Gao[6], Junyi Yin[7], Lanlin Zhang[8], Boning Ma[9], Zhiqian Hu[3,*], Pengfei Zhang[8,10,*], Hong Jiang[7,*]

[1]State Key Laboratory of Oncology in South China, Cancer Center, Collaborative Innovation Center for Cancer Medicine, School of Life Sciences, Sun Yat-sen University, Guangzhou 510060, Guangdong, P. R. China

[2]Department of Cellular and Molecular Diagnostics Center, Sun Yat-sen Memorial Hospital, Sun Yat-sen University, Guangzhou 510300, Guangdong, P. R. China

[3]Department of General Surgery, Tongji Hospital, School of Medicine, Tongji University, Shanghai 200092, P. R. China

[4]Department of Gastrointestinal Surgery, the First Affiliated Hospital, Zhejiang University School of Medicine, Hangzhou 310003, Zhejiang, P. R. China

[5]Department of Oncology, The First Affiliated Hospital, Jinan University, Guangzhou 510630, Guangdong, P. R. China

[6]Department of General Surgery, Shanghai General Hospital, Shanghai Jiao Tong University School of Medicine, Shanghai 200080, P. R. China

[7]Department of Cancer Center, Tongji Hospital, Tongji University School of Medicine, Shanghai 200065, P. R. China

[8]Department of Medical Oncology, Zhongshan Hospital, Fudan University, Shanghai 200032, P. R. China

[9]Zhongshan School of Medicine, Sun Yat-sen University, Guangzhou 510080, Guangdong, P. R. China

[10]Department of Medical Oncology, Shanghai Geriatric Medical Center, Shanghai 201104, P. R. China

†Zhixiang Zuo, Jiaqi Liang, Li-Na He, Kai Xu, and Muren Hu contributed equally to this study.

*Correspondence:

jianghong09123@126.com (Hong Jiang)

zuozhx@sysucc.org.cn (Zhixiang Zuo)

huzhiq163@163.com (Zhiqian Hu)

zhang.pengfei@zs-hospital.sh.cn (Pengfei Zhang)

This file includes:

Supplementary Figure S1 to S7, legends for Supplementary Figure S1 to S7, as well as Supplementary Table S1 to S3.

Supplementary Figure S1. Quality control and identification of cell sub-clusters from scRNA-seq.

Supplementary Figure S2. High-confidence malignant cells were identified from the epithelial cell cluster.

Supplementary Figure S3. Cell plasticity during GC progression.

Supplementary Figure S4. CEBPB enhances GC cell proliferation, invasion, and migration in vitro.

Supplementary Figure S5. Interaction landscape of tumor microenvironment (TME).

Supplementary Figure S6. CD155-TIGIT interaction induced $CD8^{+}$ T cell exhaustion in vitro.

Supplementary Figure S7. Validation of CEBPB-regulated CD155 expression in the mouse MFC cell by western blotting.

Supplementary Table S1. Clinical characteristics of gastric cancer patients profiled in this study.

Supplementary Table S2. Antibodies used in this study.

Supplementary Table S3. List of liver-specific genes containing CEBPB-binding enhancers.

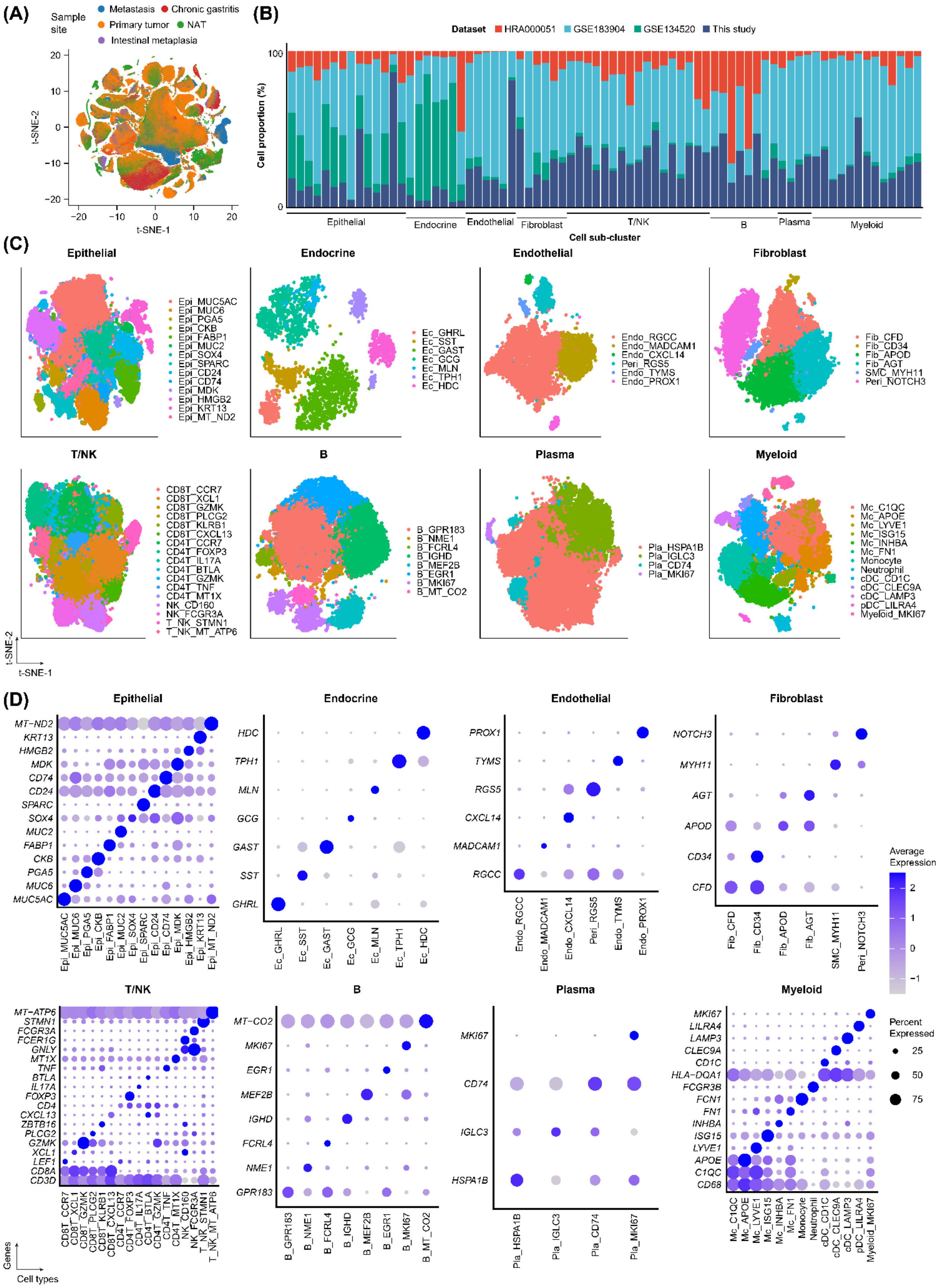

Supplementary Figure S1. Quality control and identification of cell sub-clusters from scRNA-seq. (A) A t-

SNE plot showing the distribution of single cells after quality control, derived from in-house and public single-cell RNA sequencing datasets. Cells are colored according to their sample site. The metastasis group includes both liver metastases and peritoneal metastases. **(B)** Proportion of cells from the original datasets for each cell sub-cluster. The annotation order of cell sub-clusters on the x-axis is identical to that in (C) within the same main cell cluster. **(C)** t-SNE embedding and cell sub-cluster assignment for each main cell cluster. **(D)** Corresponding marker gene expression of each cell sub-cluster. Mast cells of the myeloid lineage were clustered and classified at the meta cell-type level, and therefore not illustrated in the myeloid lineage in (C) and (D). Abbreviations: Ec, endocrine; Endo, endothelial; Epi, epithelial; Fib, fibroblast; Mc, macrophage; NAT, normal adjacent tissue; Peri, pericyte; Pla, plasma; SMC, smooth muscle cell; t-SNE, t-distributed stochastic neighbor.

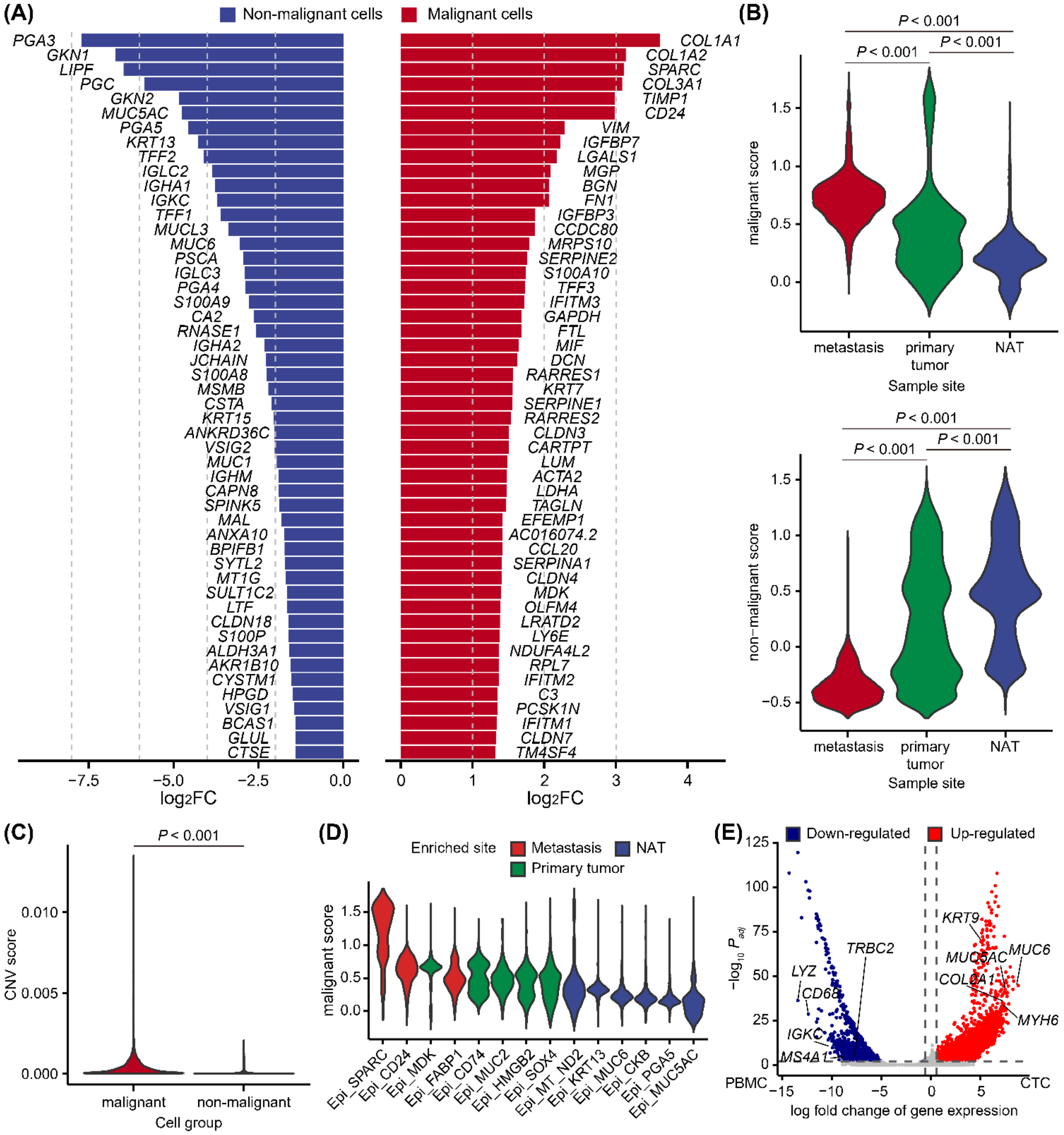

Supplementary Figure S2. High-confidence malignant cells were identified from the epithelial cell cluster. **(A)** Top 50 differentially expressed genes between malignant and non-malignant cells. **(B)** Malignant (top) and non-malignant (bottom) expression scores of epithelial cells originated from metastases, primary tumors, and NATs. **(C)** Inferred CNV scores of malignant and non-malignant cells. Only cells with high-quality CNV profiles (No. of unique molecular identifier > 3000) were included. **(D)** Malignant scores for each epithelial cell cluster. **(E)** Differentially expressed genes between CTCs and PBMCs. Up-regulated and down-regulated genes of CTCs were colored in red and blue, respectively. Significantly differentially expressed genes were identified using the thresholds (indicated by grey dashed lines) of |fold change| > 1.5 and P_{adj} < 0.01. Abbreviations: CNV, copy number variation; CTC, circulating tumor cell; PBMC, peripheral blood mononuclear cell; Epi, epithelial; FC, fold change; NAT, normal adjacent tissue.

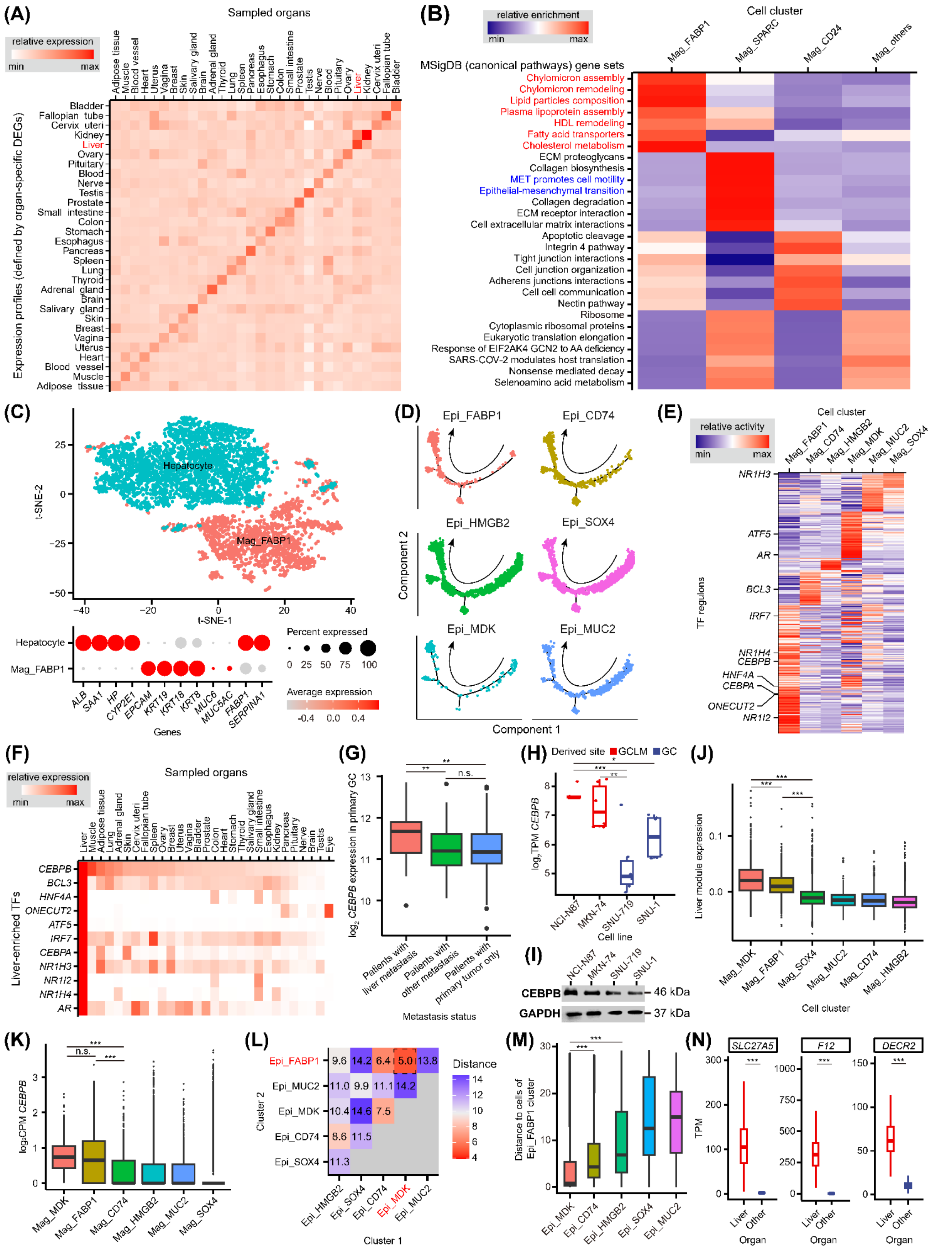

Supplementary Figure S3. Cell plasticity during GC progression. (A) Organ-specific module expression specificity across samples from various organs in the GTEx database. **(B)** Relative enrichment of functional

pathways for epithelial subpopulations. Gene sets were obtained from MSigDB database. Liver-associated gene sets are highlighted in red, and EMT-related gene sets are highlighted in blue. **(C)** Integrated analysis of malignant cell cluster Mag_FABP1 and hepatocytes from dataset GSE115469, including the t-SNE embedding (top) and the expression of the marker genes of hepatocytes and Mag_FABP1 (bottom). **(D)** Locations of cells from different epithelial cell clusters along the pseudotime trajectory inferred by the Monocle2 toolkit. **(E)** Inferred TF activity of cells from different epithelial cell clusters. The regulons of the 11 liver-enriched TFs were annotated. **(F)** Relative expression of the 11 liver-enriched TFs shown in Figure 2E across different organs. **(G)** *CEBPB* expression in primary tumor samples from GC patients with liver metastasis, with metastases other than liver, and no metastases. **(H)** *CEBPB* expression (TPM) in liver-metastatic cell lines (NCI-N87 and MKN-74; red) and non-metastatic cell lines (SNU-719 and SNU-1; blue). **(I)** CEBPB expression in NCI-N87, MKN-74, SNU-719 and SNU-1 validated by Western blotting. **(J)** Liver module expression across different cell clusters. **(K)** *CEBPB* expression (CPM) across different cell clusters. **(L)** Similarity of epithelial subpopulations evaluated by the average Euclidean distance (annotated in the cells) between different pairs of cell clusters. The numbers in the cells represent the average Euclidean distance. **(M)** Boxplot showing trajectory similarity of Epi_FABP1 with other cell clusters, evaluated by Euclidean distance. **(N)** *SLC27A5*, *F12* and *DECR2* expression (TPM) in liver versus other organs in the GTEx database. Statistical significance, *P < 0.05, **P < 0.01, ***P < 0.001. Abbreviations: ATF5, activating transcription factor 5; AR, androgen receptor; BCL3, BCL3 transcription coactivator; CEBPA, CCAAT enhancer binding protein alpha; CEBPB, CCAAT enhancer binding protein beta; CPM, counts per million; DECR2, 2,4-dienoyl-CoA reductase 2; F12, coagulation factor XII; GC, gastric cancer; GCLM, gastric cancer liver metastasis; HNF4A, hepatocyte nuclear factor 4 alpha; IRF7, interferon regulatory factor 7; Mag, malignant; NR1H3, nuclear receptor subfamily 1 group H member 3; NR1H4, nuclear receptor subfamily 1 group H member 4; NR1I2, nuclear receptor subfamily 1 group I member 2; ONECUT2, one cut homeobox 2; SLC27A5, solute carrier family 27 member 5; TF, transcription factor; t-SNE, t-distributed stochastic neighbor; TPM, transcripts per million.

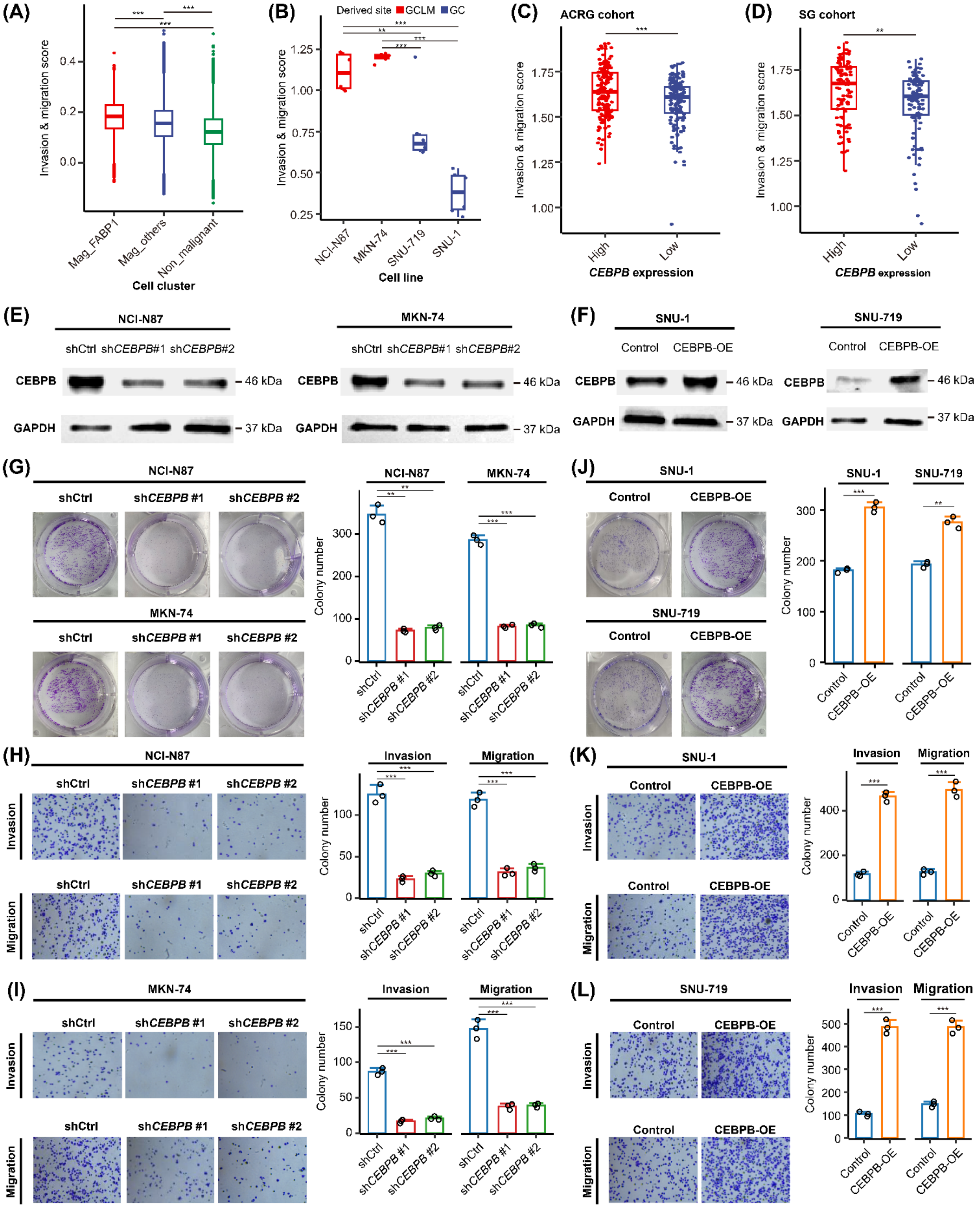

Supplementary Figure S4. CEBPB enhances GC cell proliferation, invasion, and migration in vitro. (A-B) Boxplots showing the invasion and migration score calculated using the ssGSEA methodology in different epithelial cell clusters (A) and gastric cancer cell lines derived from GCLM (red) and primary GC (blue; B). **(C-D)** Boxplots showing the invasion and migration score in patients with high or low *CEBPB* expression in the ACRG (C) and SG cohorts (D). Groups were stratified by the median *CEBPB* expression. **(E)** Validation of *CEBPB*

knockdown by shRNA in NCI-N87 and MKN-74. **(F)** Validation of *CEBPB* overexpression by lentivirus vectors in SNU-1 and SNU-719. **(G)** Effect of *CEBPB* knockdown on the proliferation ability of liver-metastatic cells NCI-N87 and MKN-74. **(H-I)** Effect of *CEBPB* knockdown on the invasion and migration abilities of NCI-N87 (H) and MKN-74 (I). **(J)** Effect of *CEBPB* overexpression on the proliferation ability of non-metastatic cells SNU-1 and SNU-719. **(K-L)** Effect of *CEBPB* overexpression on the invasion and migration abilities of SNU-1 (K) and SNU-719 (L). Data were presented as the mean ± standard deviation. Statistical significance was determined by a *t*-test. Statistical significance, *$P < 0.05$, **$P < 0.01$, ***$P < 0.001$. Abbreviations: CEBPB, CCAAT enhancer binding protein beta; GAPDH, glyceraldehyde-3-phosphate dehydrogenase; GC, gastric cancer; GCLM, gastric cancer liver metastasis; OE, overexpression; shCEBPB, CEBPB knockdown by shRNA.

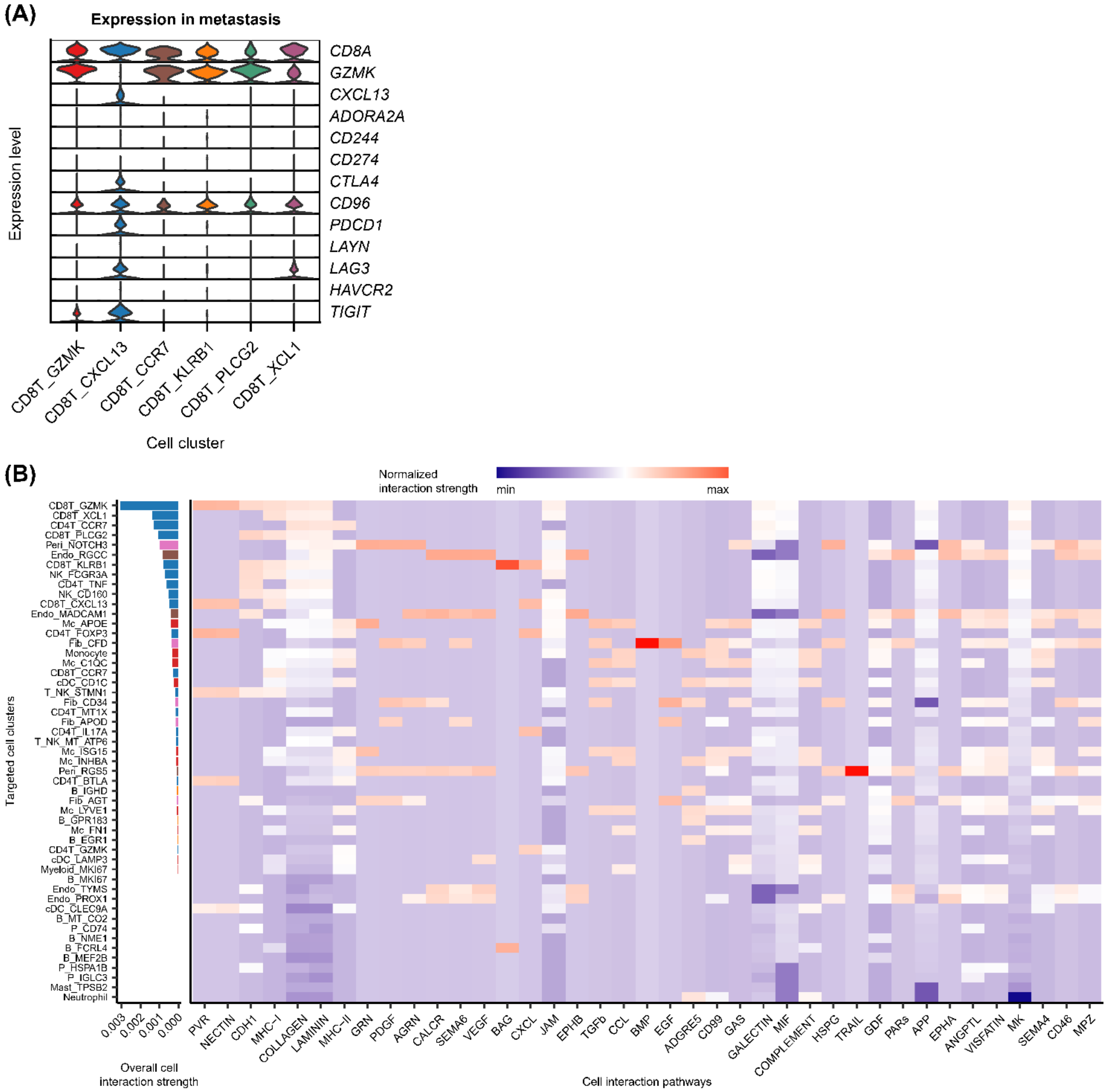

Supplementary Figure S5. Interaction landscape of tumor microenvironment. (A) Expression of marker genes and immune-suppressive-related receptors in each CD8⁺ T cell cluster in metastases. **(B)** Overall interaction strength between Mag_FABP1 and other TME cell sub-clusters (left panel) and interaction pathway landscape between Mag_FABP1 and other TME cell components in metastases (right panel, normalized between cell clusters). Abbreviations: ADGRE5, adhesion G protein-coupled receptor E5; AGRN, agrin; ANGPTL, angiopoietin-like; APP, amyloid precursor protein; BAG, BCL2-associated athanogene; BMP, bone morphogenetic protein; CALCR, calcitonin receptor; CCL, chemokine (C-C motif) ligand; CD46, cluster of differentiation 46; CD99, cluster of differentiation 99; CDH1, cadherin 1; CXCL, C-X-C motif chemokine ligand; EGF, epidermal growth factor; EPHA, EPH receptor A; EPHB, EPH receptor B; GAS, growth arrest-specific; GDF, growth differentiation factor; GRN, granulin precursor; HSPG, heparan sulfate proteoglycan; JAM, junctional adhesion molecule; MHC, major histocompatibility complex; MIF, macrophage migration inhibitory factor; MK, midkine; MPZ, myelin protein zero; PARs, protease-activated receptors; PDGF, platelet-derived growth factor; PVR, poliovirus receptor; SEMA4, semaphorin 4; SEMA6, semaphorin 6; TGFb, transforming growth factor beta;

TRAIL, TNF-related apoptosis-inducing ligand; VEGF, vascular endothelial growth factor.

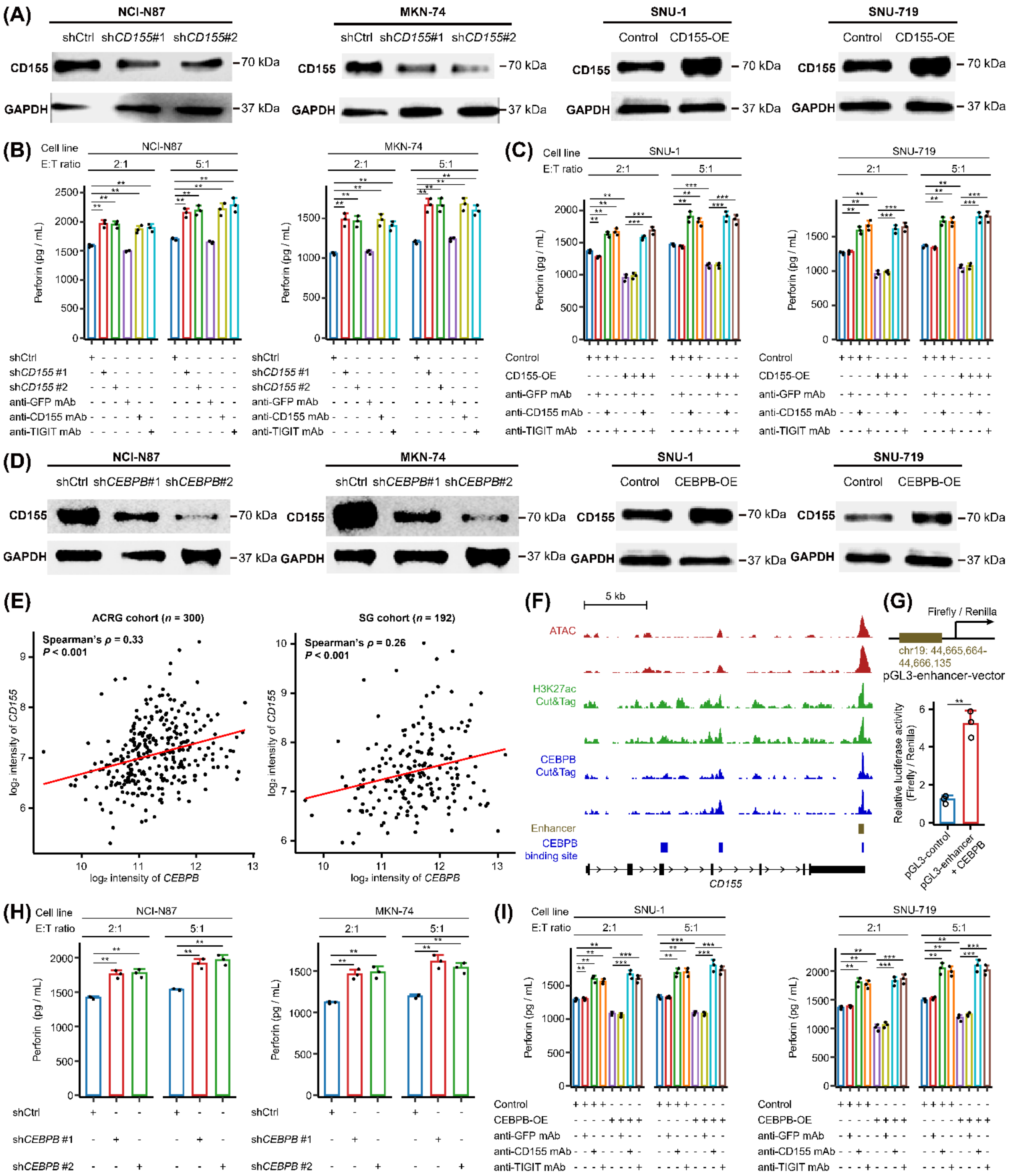

Supplementary Figure S6. CD155-TIGIT interaction induced CD8⁺ T cell exhaustion in vitro. (A) Validation of CD155 knockdown by shRNA in NCI-N87 and MKN-74, and CD155 overexpression by lentivirus vectors in SNU-1 and SNU-719, as assessed by Western blotting. **(B)** Levels of perforin secreted by $CD8^+$ T cells co-cultured with liver-metastatic gastric cancer cells under different treatments. **(C)** Levels of perforin secreted by $CD8^+$ T cells co-cultured with non-metastatic gastric cancer cells under different treatments. **(D)** Expression of CD155 after CEBPB knockdown by shRNA in NCI-N87 and MKN-74, and after CEBPB overexpression by lentivirus vectors in SNU-1 and SNU-719, as profiled by Western blotting. **(E)** Correlation analysis between the expression of *CEBPB* and *CD155* in the ACRG cohort (left) and SG cohort (right). Trend lines (red) were fitted

by linear regression. **(F)** ATAC-seq, H3K27ac Cut&Tag and CEBPB Cut&Tag signal levels of two NCI-N87 replicates, along with putative enhancer and CEBPB binding regions in the *CD155* gene locus. **(G)** Validation of candidate *CD155* enhancer by the luciferase reporter system. **(H)** Levels of perforin secreted by $CD8^+$ T cells was increased when co-cultured with CEBPB knockdown liver-metastatic gastric cancer cells. **(I)** Levels of perforin secreted by $CD8^+$ T cells was decreased when co-cultured with CEBPB overexpressing non-metastatic gastric cancer cells. Such effect could be reversed by the addition of anti-CD155 or anti-TIGIT mAbs. Data were presented as the mean ± standard deviation. Statistical significance, **P* < 0.05, ***P* < 0.01, ****P* < 0.001. Abbreviations: ACRG, Asian Cancer Research Group; ATAC-seq, assay for transposase-accessible chromatin using sequencing; CEBPB, CCAAT enhancer binding protein beta; Cut&Tag, cleavage under targets and tagmentation; E:T, effector:target ratio; mAb, monoclonal antibody; OE, overexpression; SG, Singapore; shCEBPB, CEBPB knockdown by shRNA.

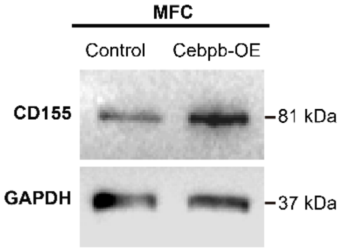

Supplementary Figure S7 Validation of CEBPB-regulated CD155 expression in the mouse MFC cell by Western blotting. Abbreviations: Cebpb, CCAAT enhancer binding protein beta; OE, overexpression.

Supplementary Table S1. Clinical characteristics of gastric cancer patients profiled in this study

ID	Age, year	Sex	In-house cohort	Sampled sites	Diagnosis	Differentiation status	TNM classification	Staging	No. of lymph node metastases (positive/all)	Liver metastasis	Single-cell analysis	No. of isolated CTCs*	IHC for CEBPB
P1	77	M	#1	T, N	adenocarcinoma with signet ring features	low-moderate	pT1N0M0	I	0	No	√	0	×
P2	61	M	#1	T, N, C	adenocarcinoma	low	pT3N3M0	IIIC	10/38	No	√	5	×
P3	63	M	#1	T, N, C	mucinous adenocarcinoma	low-moderate	pT3N1M0	IIIA	1/13	No	√	2	×
P4	68	M	#1	T, N	adenocarcinoma	low-moderate	pT3N3M0	IIIC	7/19	No	√	0	×
P5	63	M	#1	T, N, C	mucinous adenocarcinoma with signet ring features	low	pT3N1M0	IIIA	1/17	No	√	2	×
P6	75	F	#1	T, N, C	tubular adenocarcinoma	low-moderate	pT3N3M0	IIIC	22/22	No	√	8	×
P7	72	M	#1	T, N, C	tubular adenocarcinoma	low-moderate	pT3N0M0	IIB	0	No	√	7	×
P8	84	M	#1	T, N	mucinous adenocarcinoma	low	pT2N0M0	IIA	0	No	√	0	×
P9	58	F	#1	T, N, C, L	adenocarcinoma	low-moderate	pT3N2M1	IV	5/19	**Yes**	√	1	√
P10	48	M	#1	T, N, C, L	tubular adenocarcinoma	low	pT3N0M1	IV	0	**Yes**	√	1	√
P11	53	F	#1	T, N, C, L	adenocarcinoma	low	cTxN+M1	IV	unknown	**Yes**	√	1	√
P12	55	M	#2	T	adenocarcinoma	low-moderate	pT4aN0M0	IIB	0	No	×	×	√
P13	68	M	#2	T	tubular and papillary adenocarcinoma	moderate	pT4aN0M0	IIB	0	No	×	×	√
P14	72	M	#2	T	adenocarcinoma	moderate	pT1N0M0	IA	0	No	×	×	√
P15	36	F	#2	T	adenocarcinoma with signet ring features	low	pT4bN3bM0	IIIC	17/24	No	×	×	√
P16	69	F	#2	T	mucinous adenocarcinoma	low	pT4bN3aM0	IIIC	8/23	No	×	×	√

P17	62	M	#2	T	adenocarcinoma with signet ring features	low	pT4aN3aM0	IIIC	9/9	No	×	×	√
P18	63	M	#2	T	mucinous adenocarcinoma	low	pT4bN3aM0	IIIC	8/17	No	×	×	√
P19	63	F	#2	T	adenocarcinoma	low-moderate	pT4aN0M0	IIB	0	No	×	×	√
P20	72	M	#2	T	adenocarcinoma	low-moderate	pT4bN1M0	IIIB	2/17	No	×	×	√
P21	77	F	#2	T	adenocarcinoma	moderate	pT3N2M0	IIIA	6/13	No	×	×	√
P22	61	M	#2	L	adenocarcinoma	moderate	rpT3N0M1	IV	0	**Yes**	×	×	√
P23	71	F	#2	L	adenocarcinoma	low	cTxN+M1	IV	unknown	**Yes**	×	×	√
P24	69	M	#2	L	adenocarcinoma	low	rpT3N0M1	IV	0	**Yes**	×	×	√
P25	58	M	#2	L	adenocarcinoma	high	cTxN+M1	IV	unknown	**Yes**	×	×	√
P26	59	M	#2	L	adenocarcinoma	low	cT4N+M1	IV	unknown	**Yes**	×	×	√
P27	74	F	#2	L	mucinous adenocarcinoma	high	cTxN0M1	IV	0	**Yes**	×	×	√
P28	56	M	#2	L	adenocarcinoma	low-moderate	rpT4N3M1	IV	13/24	**Yes**	×	×	√
P29	75	F	#2	L	adenocarcinoma	moderate	rpT3N1M1	IV	1/26	**Yes**	×	×	√
P30	49	M	#2	L	adenocarcinoma	moderate	rpT4N1M1	IV	1/43	**Yes**	×	×	√
P31	48	M	#2	L	adenocarcinoma	low	rpT2N0M1	IV	0	**Yes**	×	×	√

*Note: a value of 0 in “No. of isolated CTCs” indicates attempted CTC enrichment but no CTCs were detected, while an “×” denotes CTC enrichment was not performed for that sample. Abbreviations: Sex: M, male; F, female. Sampled sites: T, tumor; N, normal adjacent tissue; C, circulating tumor cell; L, liver metastasis. TNM stage: prefixes 'p,' 'c,' and 'r' represent 'pathological data,' 'clinical assessment data,' and 'staging at the time of retreatment or recurrence due to disease progression,' respectively. “N+” indicates unresectable tumor cases with positive lymph node metastasis, where the specific N stage cannot be determined due to an unknown number of positive lymph nodes. IHC, immunohistochemistry; CEBPB, CCAAT enhancer-binding protein beta; CTC, circulating tumor cell.

Supplementary Table S2. Antibodies used in this study

Antibody name	Application	Dilution ratio	Manufacturer	Catalog number
Anti-Human CEBPB	IHC	1:50	Abcam	ab52194
Anti-Mouse CEBPB	IHC	1:50	Abcam	ab32358
Anti-Rabbit IgG H&L (HRP-conjugated)	IHC	1:20,000	Abcam	ab205718
Anti-Human CEBPB	WB	1:1,000	Abcam	ab244103
Anti-Human CD155	WB	1:1,000	Abcam	ab267788
Anti-Human GAPDH	WB	1:1,000	Abcam	ab8245
Anti-Rabbit IgG H&L (HRP-conjugated)	WB	1:3,000	Shanghai YbioTech	ybC-0029
Anti-Mouse IgG H&L (HRP-conjugated)	WB	1:5,000	Abcam	ab6789
Anti-Mouse CD155	WB	1:1,000	Abcam	ab103630
Anti-Mouse GAPDH	WB	1:1,000	Abcam	ab8245
Anti-Human Pan-cytokeratin	mIF	1:100	Abcam	ab234297
Anti-Human CD8	mIF	1:100	Abcam	ab237709
Anti-Human CD155	mIF	1:100	Abcam	ab267788
Anti-Human TIGIT	mIF	1:100	Abcam	ab243903
Anti-Mouse CD8	mIF	1:100	Cell Signaling Technology	98941S
Anti-Mouse IFN-γ	mIF	1:100	AiFang Biological	AF02446
Anti-Rabbit IgG H&L (HRP-conjugated)	mIF	1:20,000	Thermo Fisher Scientific	31460
Anti-Human CD155 (NTX-1088)	antagonist	-	MedChemExpress	HY-P990641
Anti-Human TIGIT (Tiragolumab)	antagonist	-	MedChemExpress	HY-P9986
Anti-GFP mAb	antagonist	-	Abcam	ab1218
Anti-Mouse TIGIT	antagonist	-	BioXCell	BE0274

Abbreviations: IHC, immunohistochemistry; mAb, monoclonal antibody; mIF, multiplex immunofluorescence; WB, Western blotting.

Supplementary Table S3. List of liver-specific genes containing CEBPB-binding enhancers

Gene symbol	Gene symbol	Gene symbol	Gene symbol	Gene symbol	Gene symbol	Gene symbol
ABAT	*AC104461.1*	*AL445493.3*	*CYP2C9*	*GNE*	*PANK1*	*SLC27A5*
ABCG8	*AC105105.2*	*AL450384.2*	*CYP4F2*	*GPT2*	*PC*	*SLC28A1*
AC004862.1	*AC106876.1*	*AL592494.1*	*DCXR*	*HAAO*	*PCK2*	*SLC2A10*
AC007221.1	*AC112206.2*	*ALDH1L1*	*DECR2*	*HNF4A*	*PCSK6*	*SLC30A1*
AC007423.1	*AC115619.1*	*ALDH2*	*EFNA1*	*IL1RAP*	*PECR*	*SLC43A1*
AC007666.1	*AC119427.1*	*ALDH4A1*	*EHHADH*	*INSIG1*	*PEMT*	*SNTB1*
AC008549.1	*AC129507.2*	*ALDH5A1*	*ELOVL6*	*ISOC2*	*PGLYRP2*	*SORD*
AC008760.2	*AC239800.2*	*ALDH6A1*	*EPB41L4B*	*IYD*	*PNKD*	*SPDYC*
AC009407.1	*ACSL1*	*ALDH8A1*	*EPHX1*	*LBP*	*POR*	*SPRYD4*
AC010280.2	*ADGRG6*	*AMBP*	*ERRFI1*	*LINC01348*	*PPP2R1B*	*STEAP3*
AC011591.1	*AL021328.1*	*ANGPTL3*	*F11R*	*LINC01485*	*PROC*	*SYT7*
AC011700.1	*AL109613.1*	*ANGPTL4*	*F12*	*LINC01843*	*PROSER2*	*TAT*
AC012065.3	*AL121827.1*	*ANGPTL6*	*F13B*	*LINC02532*	*PTGR1*	*TFR2*
AC012313.9	*AL121992.3*	*ANGPTL8*	*FAH*	*LINC02754*	*PTPN3*	*TLCD4*
AC018467.1	*AL133419.1*	*ANXA10*	*FETUB*	*LIPC*	*PXMP2*	*TM4SF4*
AC020978.4	*AL135924.2*	*ASGR1*	*FGF21*	*LPIN2*	*RDH16*	*TMPRSS6*
AC021074.3	*AL138826.1*	*BDH1*	*FGGY*	*LRRC3*	*RIDA*	*TRIB3*
AC022101.1	*AL157373.2*	*BOK*	*FST*	*MARC2*	*SAA2*	*TRPM8*
AC022167.4	*AL161668.4*	*C5*	*FTCD*	*MAT1A*	*SARDH*	*TTC39C*
AC027117.2	*AL161669.1*	*C8G*	*GAS2*	*MPST*	*SCARB1*	*UPB1*
AC079360.1	*AL161740.1*	*CLDN14*	*GDA*	*NADK2*	*SERPIND1*	*UROC1*
AC090772.1	*AL354707.1*	*COLEC10*	*GGH*	*NAGS*	*SHFL*	*XDH*
AC097639.1	*AL354872.2*	*CP*	*GLS2*	*NGEF*	*SHMT1*	*XYLB*
AC099509.1	*AL360181.1*	*CRYL1*	*GLTPD2*	*NR1I2*	*SLC15A1*	
AC099684.2	*AL391095.2*	*CYP1A2*	*GLYAT*	*OSGIN1*	*SLC22A18*	

Note: genes were selected based on their liver-specific expression and the presence of CEBPB-binding enhancers. These criteria are described in the Methods sections "Organ-specific module expression analysis" and "Identification of enhancers and their activation levels," respectively.
